# Precise staging of beetle horn formation in *Trypoxylus dichotomus* reveals the pleiotropic roles of *doublesex* depending on the spatiotemporal developmental contexts

**DOI:** 10.1371/journal.pgen.1008063

**Published:** 2019-04-10

**Authors:** Shinichi Morita, Toshiya Ando, Akiteru Maeno, Takeshi Mizutani, Mutsuki Mase, Shuji Shigenobu, Teruyuki Niimi

**Affiliations:** 1 Division of Evolutionary Developmental Biology, National Institute for Basic Biology, 38 Nishigonaka, Myodaiji, Okazaki, Japan; 2 Department of Basic Biology, School of Life Science, SOKENDAI (The Graduate University for Advanced Studies), 38 Nishigonaka, Myodaiji, Okazaki, Japan; 3 Mammalian Genetics Laboratory, Genetic Strains Research Center, National Institute of Genetics, Mishima, Shizuoka, Japan; 4 Graduate School of Bioagricultural Sciences, Nagoya University, Chikusa, Nagoya, Japan; 5 NIBB Core Research Facilities, National Institute for Basic Biology, 38 Nishigonaka Myodaiji, Okazaki, Japan; New York University, UNITED STATES

## Abstract

Many scarab beetles have sexually dimorphic exaggerated horns that are an evolutionary novelty. Since the shape, number, size, and location of horns are highly diverged within Scarabaeidae, beetle horns are an attractive model for studying the evolution of sexually dimorphic and novel traits. In beetles including the Japanese rhinoceros beetle *Trypoxylus dichotomus*, the sex differentiation gene *doublesex* (*dsx*) plays a crucial role in sexually dimorphic horn formation during larval-pupal development. However, knowledge of when and how *dsx* drives the gene regulatory network (GRN) for horn formation to form sexually dimorphic horns during development remains elusive. To address this issue, we identified a *Trypoxylus*-ortholog of the sex determination gene, *transformer* (*tra*), that regulates sex-specific splicing of the *dsx* pre-mRNA, and whose loss of function results in sex transformation. By knocking down *tra* function at multiple developmental timepoints during larval-pupal development, we estimated the onset when the sex-specific GRN for horn formation is driven. In addition, we also revealed that *dsx* regulates different aspects of morphogenetic activities during the prepupal and pupal developmental stages to form appropriate morphologies of pupal head and thoracic horn primordia as well as those of adult horns. Based on these findings, we discuss the evolutionary developmental background of sexually dimorphic trait growth in horned beetles.

## Author summary

Beetles in the family Scarabaeidae have various types of horns on their heads and thoraces, and the shape, size, number, and location of these horns are highly diversified within the group. In addition, many scarab beetle horns are sexually dimorphic. The acquisition of these evolutionarily novel horns, and the mechanisms for the diversification of these structures is an interesting question. To address this question, we focused on the rhinoceros beetle *Tripoxylus dichotomus*. Here we identified the exact developmental timepoints during which the morphological sexual dimorphism of horn primordia appears, estimated the onset of the developmental program for sexually dimorphic horn formation driven by *doublesex*, and revealed that *doublesex* regulates different aspects of cell activities during horn formation depending on particular spatiotemporal developmental contexts. Our study provides insights into regulatory shifts in these mechanisms during the evolution of sexually dimorphic traits in horned beetles.

## Introduction

Beetle horns are used as weapons for intraspecific combats between males. Beetle horns display sexual dimorphism in many Scarab species, and their shapes, numbers, sizes and forming regions are highly diverged even among closely related species [1–3]. Interestingly, the diversified horn forms are associated with the fighting styles employed by the beetles, such as scooping up, piercing and throwing [4]. Furthermore, beetle horns are thought to be an evolutionary novelty. Horns are an outgrowth structure derived not from an appendage but from a dorsal epidermal sheet. Elucidating how these novel traits were acquired in Scarab species will lead to better understanding the mechanisms of morphological diversification during evolution. Therefore, beetle horns are an attractive model for studying not only the association of trait novelty with sexually dimorphic development but also the evolution of novel traits.

**Fig 1:**
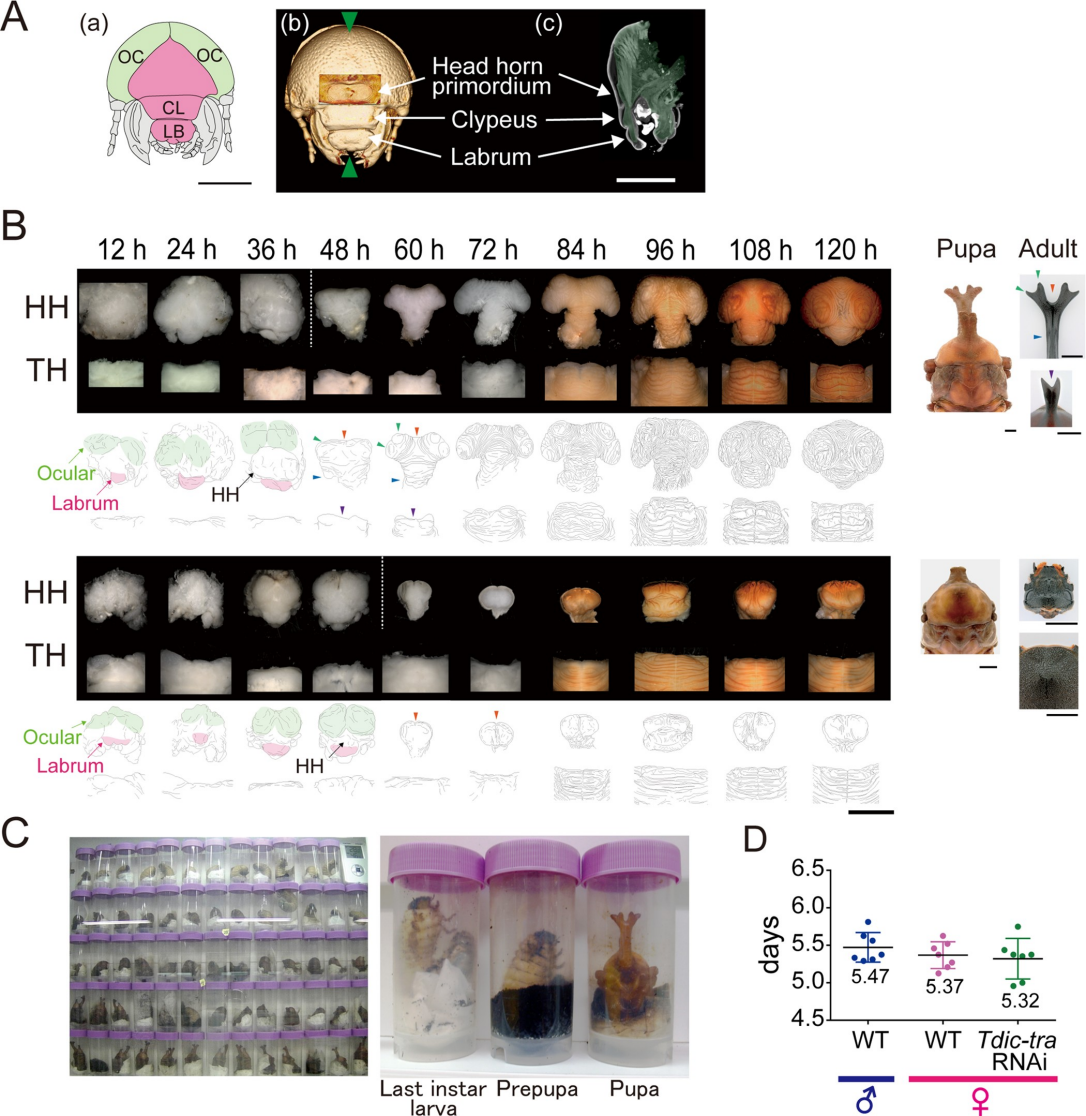
Morphological change of horn primordia and the prepupal period in *Trypoxylus dichotomus*. (A) (a) Schematic of the frontal view of larval head from *T*. *dichotomus*. Green, ocular (OC); Magenta, clypeolabrum; CL, clypeus; LB, labrum. (b), (c) Micro-CT images of prepupal head at 24 h APF from *T*. *dichotomus*. (b) The frontal view. Green arrowheads indicate the position of a sagittal section in (c). (c) The sagittal section of head. Head horn primordia were formed above the clypeus in the clypeolabrum (S1 Movie). (B) Morphological changes of head and thoracic horn primordia during larval-pupal development revealed by the time-lapse photography system. Both in males (the upper panels in the upper half) and in females (the upper panels in the lower half), horn primordia were formed in the clypeolabrum. In males, the frontal views are presented concerning the head horn primordia from 12 h APF to 36 h APF, and the anterodorsal views are presented concerning the head horn primordia from 48 h APF to 120 h APF. In females, the frontal views are presented demonstrated the head horn primordia from 12 h APF to 48 h APF, and the anterodorsal views are presented demonstrating the head horn primordia from 60 h APF to 120 h APF. The dorsal views are presented demonstrating thoracic horn primordia. APF, after pupal-chamber formation; HH, head horn primordia; TH, thoracic horn primordia; Orange arrowheads, the firstly bifurcated distal tips of a head horn; Green arrowheads, the secondly bifurcated distal tips of a head horn; Blue arrowheads, the long column shaped stalk of a head horn; Purple arrowheads, the bifurcated distal tips of a thoracic horn. Scale bars are 5 mm. (C) Snapshot images from time-lapse photography (S2 Movie). The panel on the left is a single raw image of time-lapse photography observing 64 larvae bottled in 100 ml plastic test tubes at the same time. The three panels on the right side are the magnified images of *T. dichotomus* at different developmental stages. (D) The durations of the prepupal periods in males and females estimated using the time-lapse photography system. Median, the upper/lower quantile, and minimum/maximum values were presented by a box- and whisker- plot. The median duration of prepupal periods in males and females were 131 hours and 129 hours, respectively. The mean duration of prepupal period in *Tdic-tra* RNAi females was 127 hours, which was slightly shorter (4 hours) than that of normally developed females.

Beetle horn development has been investigated in several horned beetles including the Japanese rhinoceros beetle, *Trypoxylus dichotomus* (Coleoptera, Scarabaeidae, Dynastinae). In *T*. *dichotomus*, male adults have sexually dimorphic exaggerated horns on the head and prothorax, which are used in combat among conspecific males as weapons [5, 6]. The head horn is shaped like a plow with a long stalk, and bifurcated twice at the distal tip, while the prothoracic horn is shorter than the head horn, and bifurcated once at the distal tip. During development, the horns are first formed as thickened epidermal primordia at the prepupal stage. Male adults have exaggerated horns at the head and prothoracic regions, whereas females do not have these structures. However, females have a small head horn at the pupal stage (Fig 1B, S1 Fig). Sexual dimorphism of horns can be first observed in horn primordia formed during the prepupal stage, and the horn primordia grow larger through cell growth before pupation [7, 8]. In males, the length of the pupal horn primordia after pupation is almost the same as that of an adult horn [9]. However, the shape of a pupal horn is rounded, and slightly larger than an adult horn (Fig 1B, S1 Fig). During the pupal period, the horn primodium is transformed to become an sophisticated adult horn shape through a process known as “horn remodeling” during which programmed cell death sculpts specific regions of the horn primordium into the adult morphology [7, 10, 11].

In insects, orthologs of a transcription factor gene *doublesex* (*dsx*) play a pivotal role in sexual differentiation. These orthologs are involved in the formation of sexually dimorphic traits through the expression of sex-specific isoforms (*dsxM* and *dsxF*) [8, 12–17]. In addition, tissue-specific expression of *dsx* is elevated in regions where sexually dimorphic structures are formed to induce their development [17–21]. In horned beetles including *T*. *dichotomus*, the *dsx* orthologs also regulate sexually dimorphic horn formation during larval-pupal development. RNA interference (RNAi) targeting *dsx* results in intersexual phenotypes both in males and females [8, 14].

The molecular mechanisms of sex-specific splicing of *dsx* has been intensely studied in *Drosophila melanogaster* [12, 22]. In *D*. *melanogaster*, *Sex-lethal* (*Sxl*), the master sex determination gene, initiates the sex determination cascade. *Sxl* encodes an RNA-binding protein that directly binds to target RNAs. Functional Sxl protein is translated only in females [23–25], and controls sex-specific splicing of *transformer* (*tra*) to produce functional Tra protein in females [26]. This TRA molecule then forms a heterodimer with a ubiquitously expressed RNA-binding protein, Transformer2 (Tra2), to regulate the sex-specific alternative splicing of *dsx* by directly binding to *dsx* transcripts in females [27, 28]. The resultant *dsxF* isoform in females and *dsxM* isoform in males regulate a battery of downstream genes to form sexually dimorphic traits by binding to the target DNA sequences with the Dsx binding motif [29, 30], and by activating or repressing their transcription [31]. *intersex* (*ix*) functions as a female-specific co-activator by directly binding to female-specific isoforms of the DsxF protein, and regulates development of female-specific traits [32].

The *Sxl*-[*tra/tra2*]-[*dsxF/ix*] pathway described above is only activated in females. In males, default mRNA splicing results in expression of the male-specific splicing variant of *dsx*, *dsxM* [12, 22]. Loss of function of the sex determination genes in females, and gain of function in males result in sex transformation [12, 22].

Orthologous genes corresponding to the *D*. *melanogaster* sex determination genes described above are conserved among many holometabolous insects. In addition, the function of splicing regulatory factors (Tra/Tra2) and a transcription co-factor (Ix) that are supposed to directly interact with *dsx* transcripts or Dsx protein are conserved in many holometabolous insects (e.g. Diptera: *Drosophila melanogaster* [33], the housefly *Musca domestica* [34], the Mediterranean fruit fly *Ceratitis capitata* [35], the Australian sheep blowfly *Lucilia cuprina* [36], the oriental fruit fly *Bactrocera dorsalis* [37], Hymenoptera: the honeybee *Apis mellifera* [38], the parasitic wasp *Nasonia vitripennis* [39], Coleoptera: the red flour beetle, *Tribolium castaneum* [40, 41]). However, whether these genes are involved in the sex determination pathways to form sexually dimorphic horns in *T*. *dichotomus* remains elusive. In addition, when and how *dsx* interacts with the gene regulatory network (GRN) for horn formation to drive cellular activities such as cell growth, cell death and cell movement is also unknown.

To understand the developmental and genetic mechanisms underlying sexually dimorphic horn formation in *T*. *dichotomus*, we first describe a precise time course of the morphogenetic changes of male and female horn primordia during larval-pupal development using time-lapse photography. Next, we examined the function of putative sex determination genes in *T*. *dichotomus* using larval RNAi by focusing on orthologs of known *D*. *melanogaster* sex determination genes. Moreover, we investigated the initiation timing of the GRN for horn formation by knocking down *Tdic-tra* at multiple developmental timepoints, and by evaluating the extent of sex transformation phenotypes. Based on these experiments, we concluded that the GRN for horn formation, which is supposed to be modified by *dsxM* and *dsxF* functions in males and females, is driven at a very early stage of larval-pupal development before clear morphological changes in horn primordia can be detected. Furthermore, we show that *dsxM* has different functions in both the prepupal and pupal stages during the formation of appropriate morphologies in pupal and adult horns in males. Based on these findings, we discuss the evolutionary developmental background of sexually dimorphic horn formation in horned beetles.

## Results

### Development of sexual dimorphism in *T*. *dichotomus* horn primordia

To identify the developmental timepoint when sexual dimorphism of horns first appears in *T*. *dichotomus*, we described morphological changes of head and thoracic horn primordia during the prepupal period. Micro-CT analysis of a head horn primordium at an early stage (24 hours after pupal-chamber formation; 24 h APF) in the prepupa revealed that a head horn primordium was formed in the clypeolabral region during the prepupal stage as investigated in *Onthophagus taurus*, *Onthophagus sagittarius* and *Onthophagus gazella* (Fig 1A and 1B, S1 Movie) [3, 42–44]. In addition, head horn primordia were formed above the clypeus in the clypeolabrum (Fig 1A, S1 Movie). To determine the exact timepoint when protrusion of the primordium is initiated, we established a time-lapse photography system (Fig 1C, S2 Movie). Until recently, developmental staging of *T*. *dichotomus* prepupae had been difficult because they form pupation chambers and pupate underground. Using our time-lapse photography system, we found that the head-rocking behavior at the end of pupal-chamber formation can be an unambiguous marker for the initiation of the prepupal stage (S2 Movie). We could minimize the developmental deviation between individuals within 10 hours using this precise developmental marker (Fig 1C, S2 Movie). The average prepupal period was 5.5 ± 0.19 days (131 ± 4.7 hours) in males and 5.4 ± 0.17 days (129 ± 4.3 hours) in females (Fig 1D). Based on this staging paradigm, we manually dissected out horn primordia every 12 h after pupal-chamber formation (APF). We found that sexual dimorphism of horn primordia appeared at 36 h APF (Fig 1B). Therefore, we concluded that the GRN driving the formation of horn sexual dimorphism would be activated before 36 h APF in *T*. *dichotomus*. In addition, we also found that apolysis occurring at 36 h APF can be another unambiguous developmental marker. Larval mandibular tendons that tightly connect mandibular muscles and apodemes (S3 Movie) [44–46] were completely detached at 36 h APF. This feature, along with the apolysis occurring at every body part including the neighboring ocular region also allowed us to know the timing of the onset of sexual dimorphism in these beetles.

### Identification of genes regulating the sex-specific splicing of *Tdic-dsx* during *T*. *dichotomus* horn formation

In *T*. *dichotomus*, the regulatory factors associated with sex-specific splicing of *dsx* had not been identified. We searched for such regulatory factors focusing on *T*. *dichotomus* orthologs of known *D*. *melanogaster* sex determination genes (*Sxl*, *tra*, *tra2* and *ix*) [12, 22].

**Fig 2:**
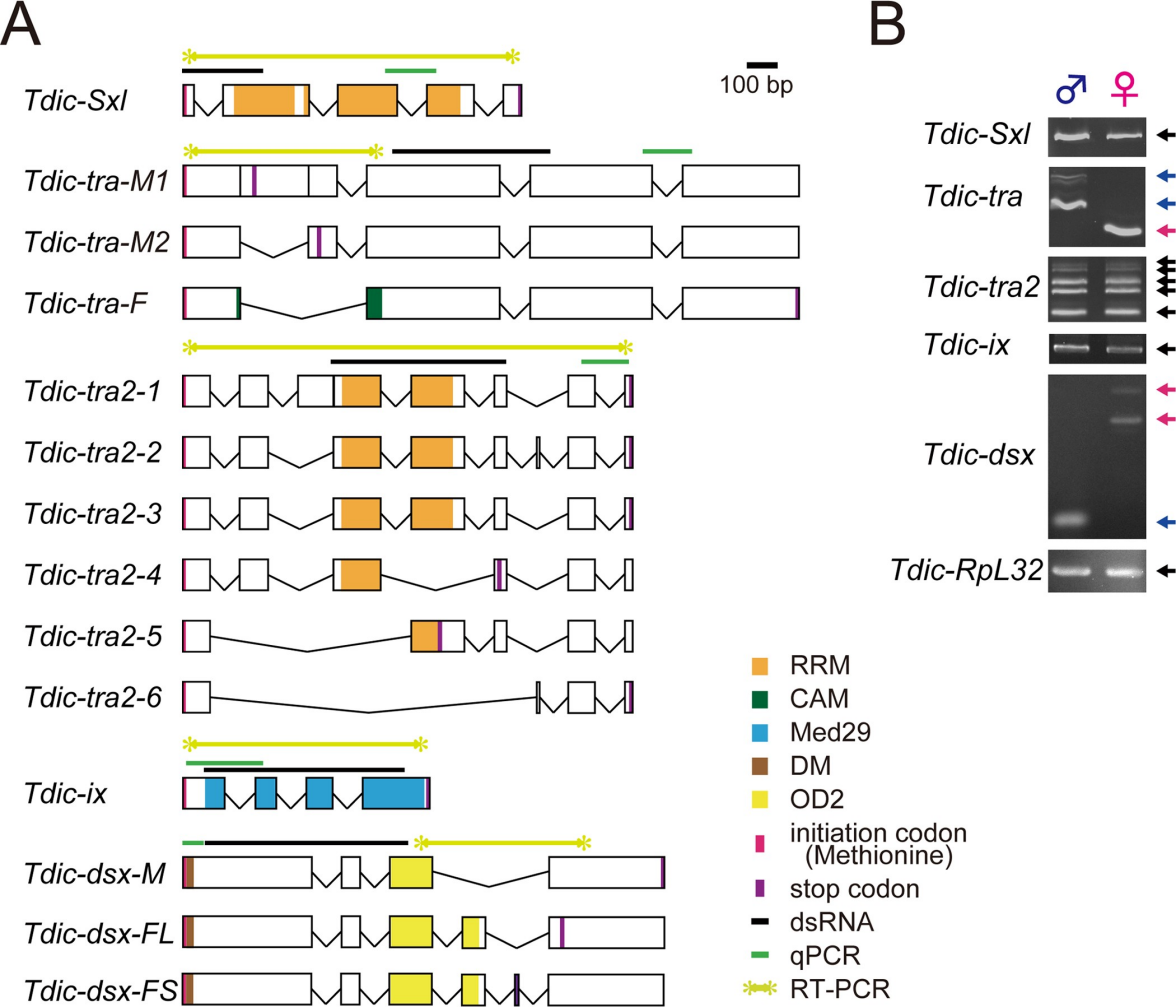
Schematic representation of putative exon–intron structures and sex-specific splicing patterns of the sex determination genes. (A) Schematics of putative exon-intron structures of the sex determination genes in *T*. *dichotomus*. Orange box, RRM (RNA binding motif); Green box, CAM (C, *Ceratitis*; A, *Apis*; M, *Musca*) domain [34, 47], which is conserved in all *tra* orthologs identified in other insects except for those in *Drosophila* species and the sandfly (*Phlebotomus papatasi*) [48]; Light blue box, Med29 (mediator complex subunit 29) domain; Brown box, DM (Doublesex/Mab-3 DNA-binding) domain; Yellow box, OD2 (Oligomerization dmain 2); Black bars, template sequences for dsRNA synthesis; Green bars, the amplified regions in qRT-PCR analysis; Light green bars, the amplified region to test sex-specific splicing variants; Magenta lines, translation start sites; Purple lines, stop codons. (B) Evaluation of expression and sex-specific splicing patterns in the sex determination genes in male and female prepupal head horn primordia. Template cDNAs were derived from prepupal horn primordia at 72 h APF. *Tdic-RpL32* was used as an internal control for RT-PCR. Blue arrows, PCR products with male specific splicing variants; Magenta arrows, PCR products with female specific splicing variants; Black arrows, PCR products amplified in both sexes.

First, we investigated whether these genes produce sex-specific splicing variants in *T*. *dichotomus* by RT-PCR. All of these genes were expressed in male and female prepupal head and thoracic horns (Fig 2A light green bars, Fig 2B, S1 Table) [47, 48]. Among these genes, sex-specific splicing variants were detected only in *Tdic-tra* (Fig 2B). Next, to test whether these genes function as sex determination genes, we performed larval RNAi experiments using dsRNA targeting the common regions between sexes (Fig 2A, black bars, S1 Table, S2 Table).

**Fig 3:**
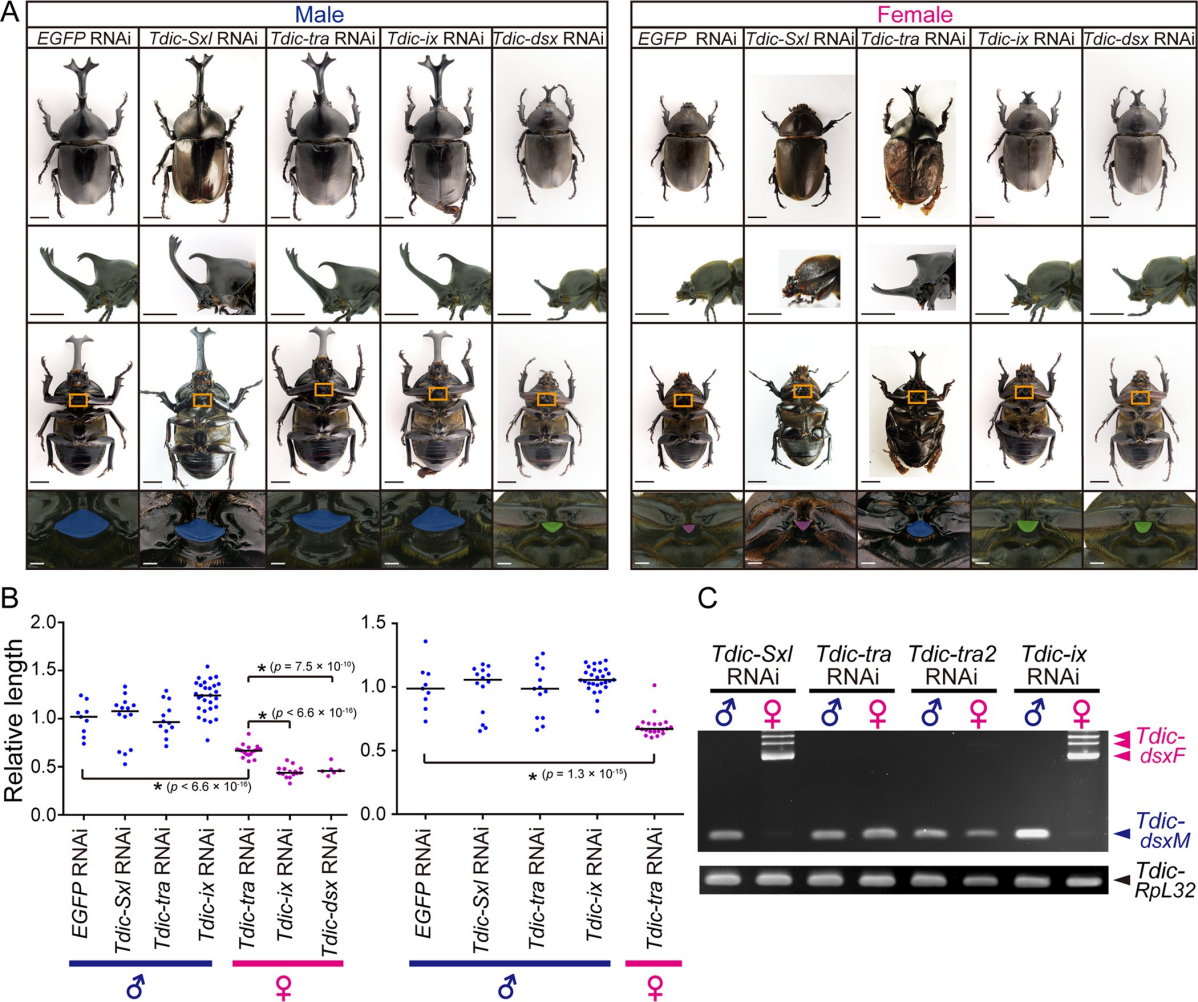
RNAi-mediated loss-of-function phenotypes of the sex determination genes. (A) Representative individuals in each RNAi treatment in males (the left half) and females (the right half). Each dsRNA was injected into last-instar larvae. The negative control RNAi treatment (*EGFP* dsRNA) showed no morphological defects. The upper row, the dorsal views of adults; the second row, the lateral views of a head and a prothorax in adults; the third row, the ventral views of adults; the forth row, magnified views of orange squares (IPP, intercoxal process of prosternum) in the third row. Scale bars are 1 cm in the upper three rows and 1 mm in the fourth row. (B) Quantification of the relative head and thoracic horn length in RNAi-treated individuals. Relative head and thoracic horn length in RNAi-treated males and females are plotted in the blue dots and the magenta dots, respectively. The relative horn lengths were standardized by dividing the horn length by the body size in each RNAi treated individual and by the mean horn length of the *EGFP* RNAi-treated males. Differences in horn length among individual RNAi treatments were compared using Brunner-Munzel test. These p-values were adjusted by the Bonferroni correction. Asterisks denote significance: n.s., p > 0.05; *, p < 0.05. (C) Sex-specific splicing of *Tdic-dsx* in RNAi treatments targeting *Tdic-Sxl*, *Tdic-tra*, *Tdic-tra2* and *Tdic-ix*. Blue arrowheads, male specific splicing patterns. Magenta arrowheads, female specific splicing patterns. *Tdic-RpL32* was used as an internal control for RT-PCR.

In females, morphological changes was observed in the RNAi treatments targeting *Tdic-tra* and *Tdic-ix*, whereas no morphological changes were observed in males (Fig 3A, S2 Fig). Such female-specific phenotypes were comparable with the mutant phenotypes of *tra*, *tra2* and *ix* in *D*. *melanogaster* [12, 22]. Concerning *Tdic-tra2*, we could not observe adult phenotypes due to the prepupal lethality of RNAi injection in both sexes. The phenotypes in *Tdic-tra* RNAi females and *Tdic-ix* RNAi females were different and the *Tdic-ix* RNAi phenotype was similar to the effects of the *Tdic-dsx* RNAi phenotype (Fig 3A). In the *Tdic-tra* RNAi females, ectopic horn formation was observed in both the head and prothorax (Fig 3A), whereas in *Tdic-ix* RNAi females, ectopic horns were formed only in the head, and these horns were significantly shorter than the ectopic head horns in *Tdic-tra* RNAi females (Fig 3A and 3B). Such a difference in morphology was also observed in another sexually dimorphic structure, the intercoxal process of the prosternum (IPP). Male IPPs are generally larger than female IPPs (Fig 3A). Although both the *Tdic-tra* and *Tdic-ix* RNAi females have larger IPPs than female controls (*EGFP* RNAi), *Tdic-tra* RNAi females have much larger IPPs than *Tdic-ix* RNAi females (Fig 3A). These results and the intermolecular interactions reported in *D*. *melanogaster* led us to predict that *Tdic-tra* may also regulate the sex-specific splicing of *Tdic-dsx* in *T*. *dichotomus*. To test this, we investigated the splicing patterns of *Tdic-dsx* in the above RNAi-treated males and females. We designed PCR primer sets to amplify the region including the whole female specific exon, which is spliced out in males (Fig 2A, light green bars, S1 Table). We found that the sex-specific splicing pattern observed in wild type (Fig 2B) was switched in *Tdic-tra* and *Tdic-tra2* RNAi treatments, whereas splicing patterns were not changed by *Tdic-Sxl* and *Tdic-ix* RNAi treatments (Fig 3C). These data indicate that *Tdic-tra* and *Tdic-tra2* regulate female-specific splicing of *Tdic-dsx* in *T*. *dichotomus*. The switching of sex-specific splicing of *Tdic-dsx* and the resultant morphological sex transformation in *Tdic-tra* RNAi females implies that the sex-specific splicing regulation of *Tdic-dsx* by *Tdic-tra* is also conserved in *T*. *dichotomus* as in other holometabolous insects investigated so far [33–41]. Taken together, we concluded that *Tdic-tra* functions as a sex determination gene during horn formation in *T*. *dichotomus*.

### Estimation of the onset of the developmental program for sexually dimorphic horn formation using *Tdic-tra* RNAi

As sexual dimorphism of horn primordia first appeared at 36 h APF (Fig 1B), we speculated that the onset of the developmental program for sexually dimorphic horn formation was initiated before 36 h APF. To estimate this timepoint more accurately, we performed *Tdic-tra* RNAi in females at multiple developmental timepoints during pupal chamber formation periods and prepupal periods, and evaluated the extent of sexual transformation in horns.

If the timing of *Tdic-tra* RNAi in females is early enough, the ectopic *Tdic-dsxM* would be expressed in female horn primordium from the onset of developmental program for sexual dimorphism formation, and full sexual transformation can be achieved. On the other hand, the later the timing of *Tdic-tra* RNAi treatment becomes, the more the initial phases of the male-specific horn formation program driven by ectopically expressed *Tdic-dsxM* are trimmed, and at the same time repressed by normally expressed *dsxF*. Therefore, by determining the latest RNAi injection timing when a full sexual transformation phenotype is observed, we can estimate the onset of the sexually dimorphic horn formation program mediated by the ectopic *Tdic-dsxM* expression.

**Fig 4:**
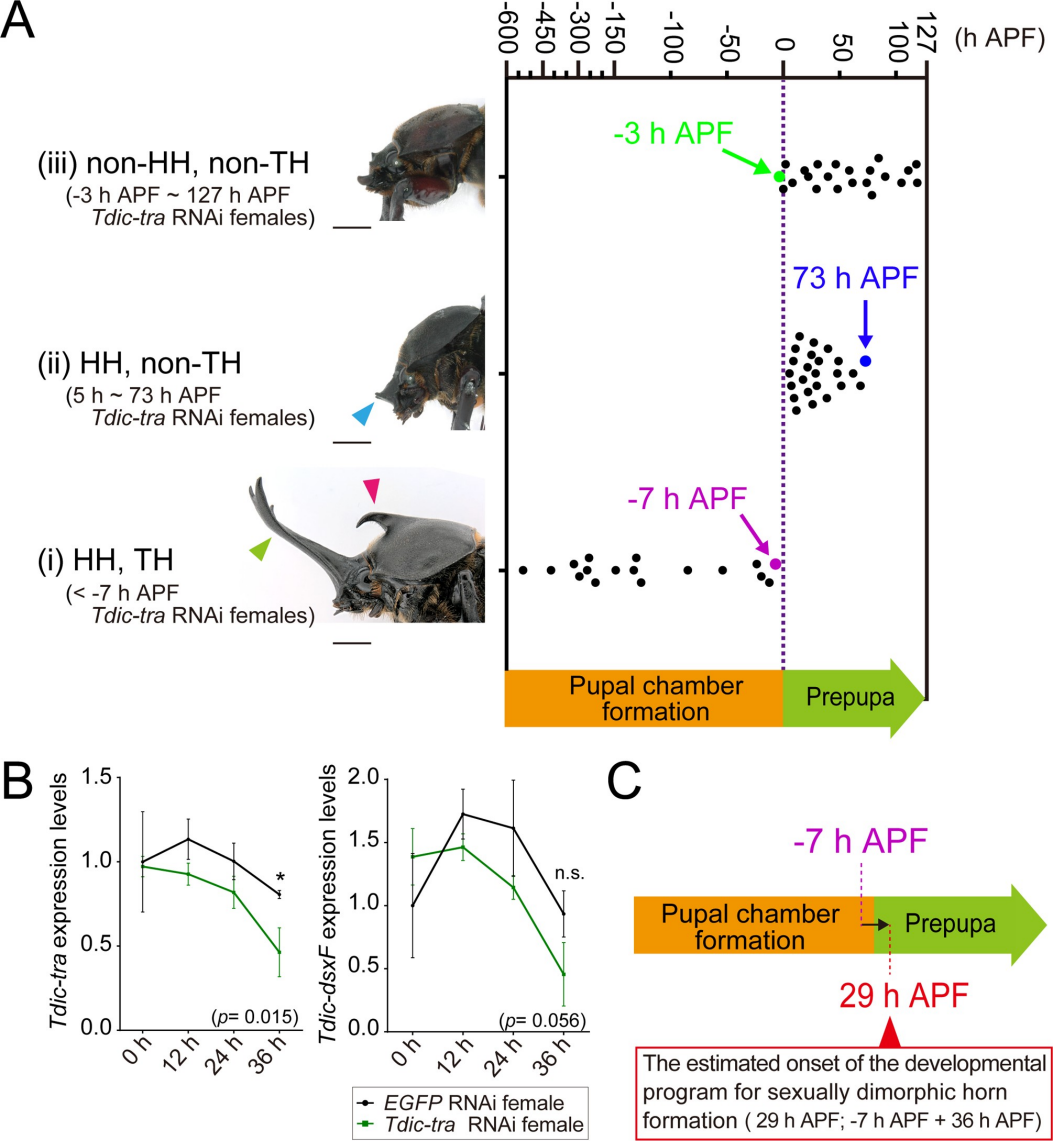
Estimation of the onset of the *Tdic*-*dsx* dependent horn developmental program by *Tdic-tra* RNAi. (A) Relationship between the timepoints of *Tdic-tra* dsRNA injection and the extent of sex transformation. The extent of the sex transformation (masculinization) phenotypes of *Tdic-tra* RNAi females were categorized into three classes: (i) prominent HH and TH were formed (< -7 h APF); (ii) only small HH was formed (5 h APF ~ 73 h APF); (iii) neither the HH nor TH was formed as in normal females (-3 h APF ~ 127h APF). HH, head horn primordia; TH, thoracic horn primordia. The dot plot panel was timepoints of *Tdic-tra* dsRNA injection before pupation in each class. The green dot, the magenta dot, and the blue dot indicate -3 h APF, -7 h APF and 73 h APF, respectively. Scale bars are 5 mm. (B) Time course of relative *Tdic-tra* and *Tdic-dsxF* mRNA expression level after *Tdic-tra* dsRNA injection at 24 h APF. mRNA expression levels at each timepoint were quantified by qRT-PCR. The expression levels of *Tdic-tra* were significantly decreased at 36 hours after injection (p<0.015). The expression level of *Tdic-dsxF* tended to be decreasing as time went on, but there were no statistically significant difference (p<0.056). (C) Summary of sex transformation phenotypes and the estimated onset of the *Tdic-dsx* dependent horn developmental program. The magenta line is the boundary timepoints of phenotype (i). Red arrowheads indicate the timepoints corrected by the mRNA degradation delay (36 hours), which estimated by qRT-PCR. APF, after puapal-chamber formation; Red arrowhead, the estimated onset of the developmental program for sexually dimorphic horn formation driven by *Tdic-dsx*.

In this experiment, fully matured female last instar larvae and unstaged female prepupae were injected with *EGFP* or *Tdic-tra* dsRNA (S3 Table). We performed time-lapse photography until pupation using these larvae, and retrospectively estimated the exact timing of injection before pupation. In addition, we estimated how many hours after pupal chamber formation (APF) each injection had been performed by subtracting the duration between each injection timepoint and pupation timepoint from the mean duration of the prepupal period in *Tdic-tra* RNAi females (127 hours) (Fig 1D). Our data indicated that *Tdic-tra* RNAi females treated earlier than the timepoint of -7 h APF formed fully developed male horns (Fig 4A(i), magenta dot). In contrast, *Tdic-tra* RNAi females treated later than the timepoint at -3 h APF showed either no morphological changes or only modest sex transformation of head or horn, if any (Fig 4A(ii) and (iii), green dot). These data suggested that the estimated timepoint of the onset of the developmental program for sexually dimorphic horn formation is around -7 h APF.

Since this timepoint was estimated by means of RNAi, we speculated that there should be time lag between the timing of injection and the timing of the decrease in functional protein levels followed by the mRNA degradation. Thus, we also quantified expression dynamics of *Tdic-tra* mRNA after RNAi treatment by qRT-PCR. It was technically impossible to monitor the expression dynamics of *Tdic*-*dsx* at -7 h APF, which is estimated to be before pupal chamber formation (Fig 1D, Fig 4A). Then, we monitored the expression dynamics as early as possible (dsRNA injection at 24 h APF), instead. The expression levels of *Tdic-tra* and *Tdic-dsxF* were quantified every 12 hours up to 36 hours after injection (Fig 2A, green bars, Fig 4B). As a result, the expression levels of *Tdic-tra* and *Tdic-dsxF* were decreased to less than half of that of the negative control at 36 hours after injection (Fig 4B). Since mRNA started to be degraded 36 hours after RNAi treatments (Fig 4B), we estimated that this timepoint corresponded to 29 h APF (−7 plus 36) (Fig 4C). Taking morphological data into account, this timepoint corresponded to 7 hours before the initial sexual dimorphism appears in horn primordia (Fig 1B, Fig 4C).

### The spatiotemporal expression pattern of *Tdic-dsx* in the head horn primordium

**Fig 5:**
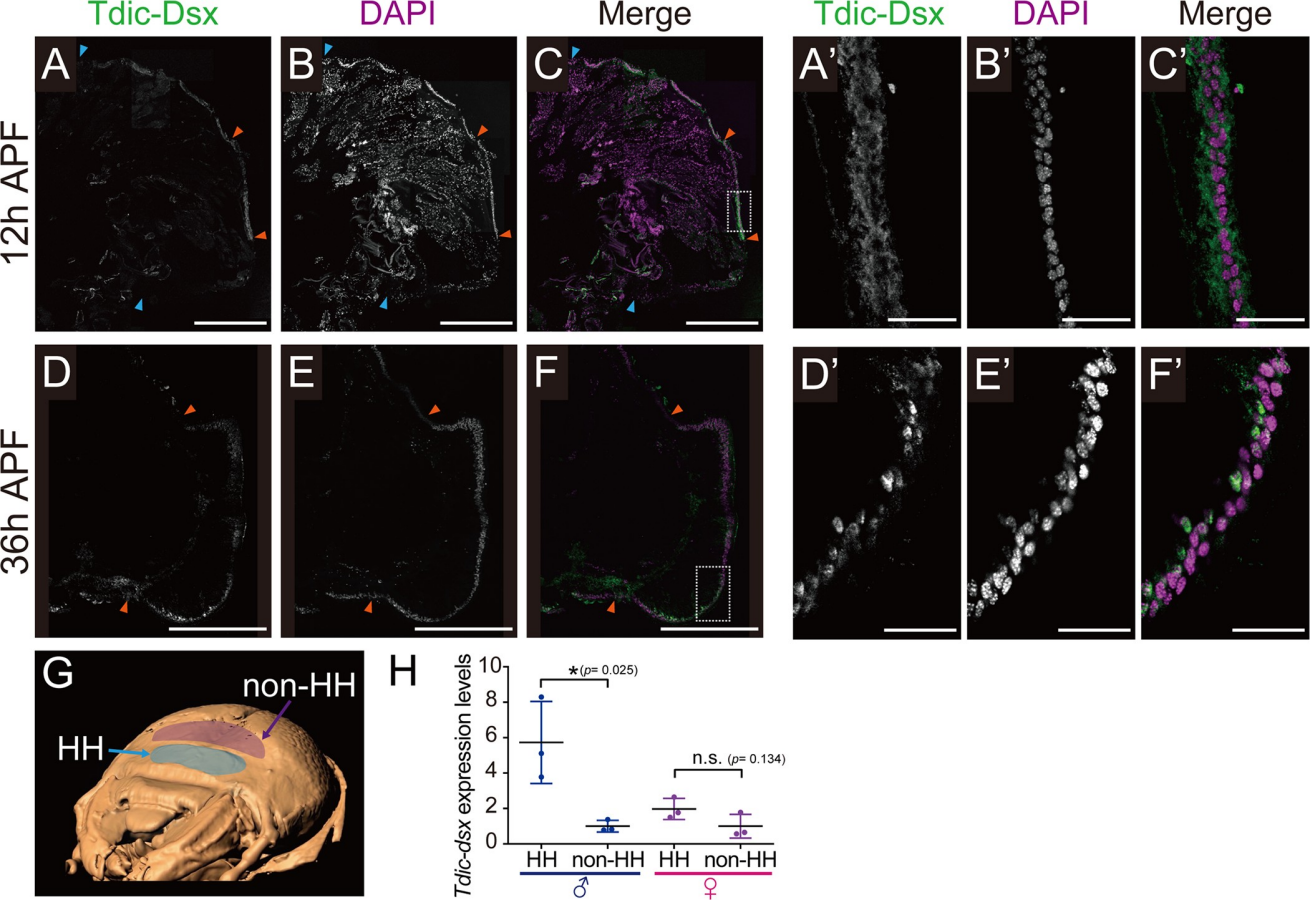
Spatial and temporal expression of Tdic-Dsx protein in the head horn primordium during larval-pupal development. (A-F) The head epidermis including the head horn primordium was stained with DAPI (magenta) to label nuclei and with anti-Tdic-Dsx antibody (green) to label Tdic-Dsx protein. Tdic-Dsx protein showed higher expression in the head primordial epidermis than in the surrounding head epidermis. Between Orange-Orange arrowheads is the head horn primordium. Between Orange-Blue arrowheads is the surrounding head epidermis. (A-C) Tdic-Dsx expression pattern in 12 h APF, Scale bars are 500 μm. (A’-C’) A higher magnification of the head horn primordium, respectively. Tdic-Dsx protein localization was cytoplasmic. Scale bars are 50 μm. (D-F) Tdic-Dsx expression pattern in 36 h APF, Scale bars are 500 μm. (D’-F’) A higher magnification of the head horn primordium, respectively. Tdic-Dsx protein localization were nuclei. Scale bars are 50 μm. (G) The epidermal regions used for qRT-PCR. The 3D volume image was reconstructed from sequential micro CT images. Blue, head horn primordium (HH); Purple, the head epidermis (non-HH). (H) The relative *Tdic-dsx* expression level in head horn primordia and head epidermis at 36 h APF in both sexes. Asterisks denote significance: n.s., p > 0.05; *, p < 0.05. HH, head horn primordium epidermis; non-HH, the head epidermis.

The expression level of Dsx protein is frequently upregulated region-specifically during development of sexually dimorphic traits in insects (e.g. Sex comb formation in *Drosophila*, mimetic wing morph formation in *Papilio*, and wing pheromone gland formation in *Bicyclus*), presumably to facilitate sexual dimorphism formation [18–21]. To test whether Tdic-Dsx protein also exhibits region-specific upregulation in the horn primordium, we raised anti-Tdic-Dsx polyclonal antibodies, and performed immunohistochemistry at the onset of sexually dimorphic horn formation (36 h APF). As a result, Tdic-Dsx protein showed higher expression in the head primordial epidermis than in the surrounding head epidermis and was mainly localized in nuclei (Fig 5D–5F and 5D’–5F’, S3 Fig). In accordance with this result, mRNA expression level of *Tdic-dsxM* were also higher in the head horn primordial epidermis than in the surrounding head epidermis (Fig 5G and 5H). In contrast, *Tdic-dsxF* did not show significantly higher expression in the horn primordium at this stage (Fig 5G and 5H). Therefore, we concluded that expression level of Dsx protein is upregulated region-specifically to form sexually dimorphic horns during development.

Interestingly, we found that Tdic-Dsx showed higher expression in the head horn primordial epidermis even before the onset of sexually dimorphic horn formation (12 h APF) (Fig 5A–5C and 5A’–5C’, S3 Fig), but the localization of expression was cytoplasmic. This finding suggests that the region-specific expression of Tdic-Dsx has been already initiated before it activates downstream genes, but its transcription factor activity is triggered only after it is translocated to nuclei.

### Sexually dimorphic morphogenesis in male and female horn primordia

**Fig 6:**
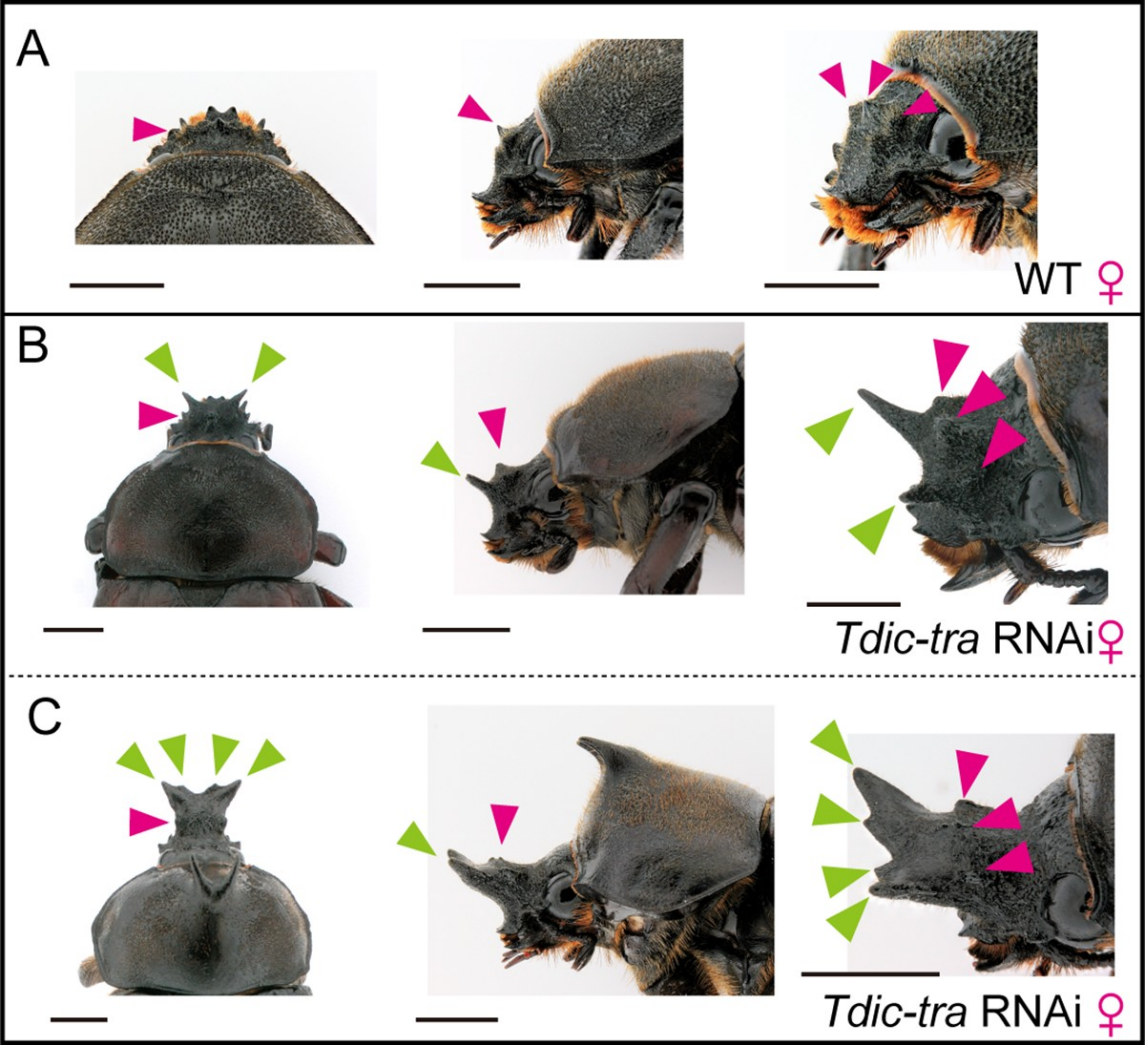
Horn formation phenotypes induced by *Tdic-tra* RNAi at late prepupal stages. (A-C) Comparison of wild type female head and ectopic intermediate sexual transformation head horn in female heads induced by *Tdic-tra* RNAi treatments. (A) A wild type female head. (B) Small ectopic head horn formation in a female head. (C) Middle-sized ectopic head horn formation in a female head. Three small protrusions are formed in clypeolabrum on the wild type female head (A-C, magenta arrowheads), whereas ectopic small- or middle-sized ectopic head horns were formed in the region anterior to the three small protrusions (B, C, green arrowheads) in *Tdic-tra* RNAi treatments. Scale bars are 5 mm.

*T*. *dichotomus* adult males have exaggerated horns at the head and prothoracic regions whereas adult females only have three small protrusions at the clypeolabral region (Fig 6A, magenta arrowhead). We focused on whether the distal tips of the male head horn and three female head protrusions were formed in the same region or whether they originated from distinct regions in the head (Fig 6A, S1 Fig). During prepupal stages, a head horn primordium in both males and females seemed to be formed in the same region (the almost entire clypeolabral region in the head) (Fig 1, S1 Fig). However, due to the lack of clear morphological landmarks indicating the formation region of male head horns and female head protrusions, within the head, the formation regions of these traits remained elusive. Unexpectedly, however, we obtained an intermediate sexual transformation phenotype of *Tdic-tra* RNAi to solve this problem. When we injected *Tdic-tra* dsRNA in small amounts (2.5 μg), we obtained several adults possessing both the three small protrusions similar to those in females, and a small ectopic anterior protrusion, seemingly analogous to a male head horn (Fig 6B and 6C). This result suggests that at least the anterior region of a male head horn is not formed from the same region as female head protrusions.

**Fig 7:**
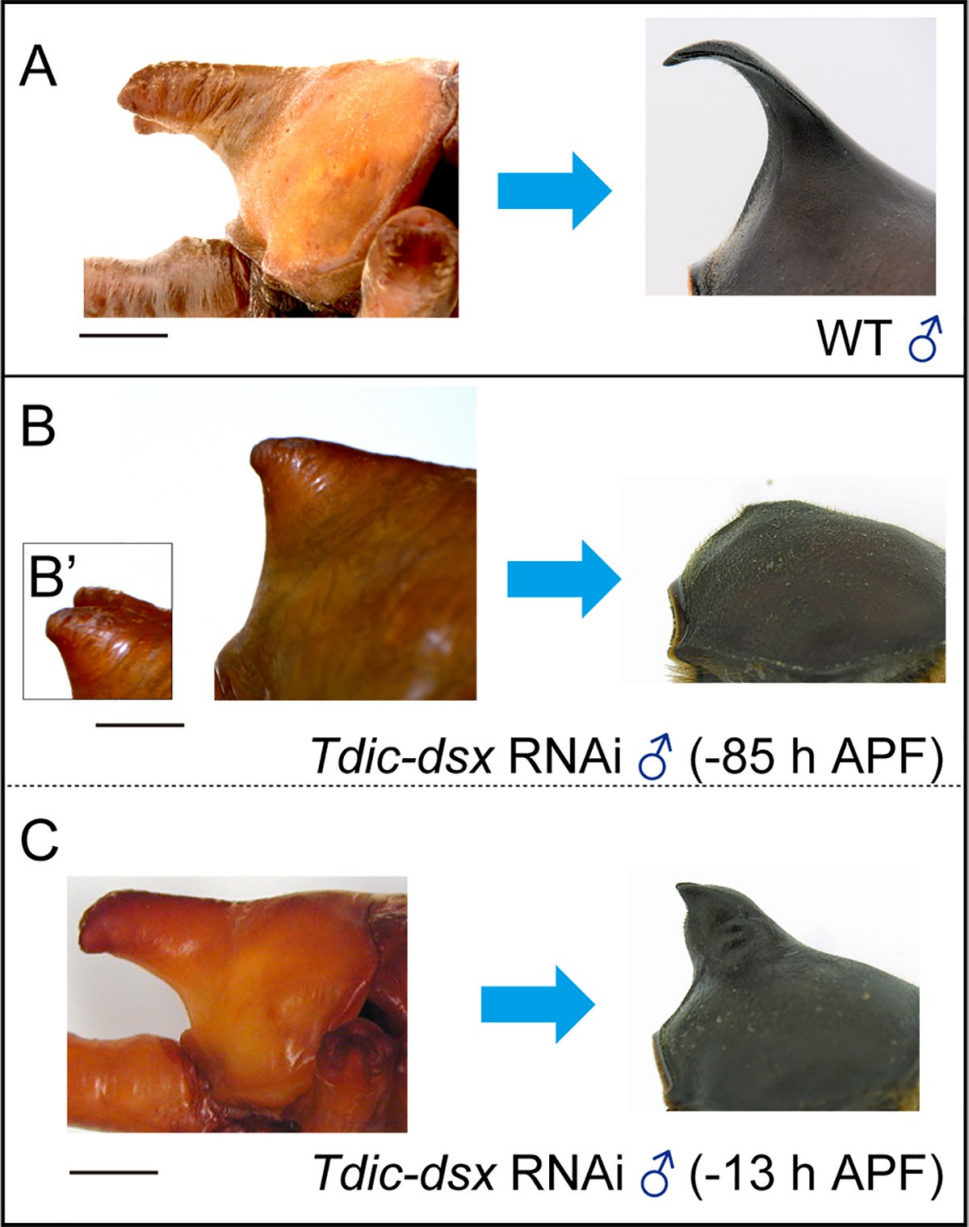
Horn formation phenotypes induced by *Tdic-dsx* RNAi at late prepupal stages. (A-C) Thoracic horn remodeling during pupal-adult development in males. (A) A wild type male thoracic horn. (B) Almost complete reduction of a thoracic horn with an early *Tdic-dsx* RNAi treatment (-85 h APF). (B’) dorsolateral view. (C) Unremodelled thoracic horn formed by a late *Tdic-dsx* RNAi treatment (-13 h APF). APF, after puapal-chamber formation. Scale bars are 5 mm.

Next, we asked whether *Tdic-dsx* regulates the entire cellular activity during sexually dimorphic horn formation. We especially focused on the two distinct developmental processes, “horn growth” before pupation, and “horn remodeling” after pupation (see details in the Introduction section). The morphological changes before and after pupation are qualitatively comparable between males and females, but the extent of the morphological changes during development is different between them. During the prepupal stage, males form longer head and thoracic pupal horns, whereas females only form smaller pupal head horns. During the pupal stage, both male head and thoracic horns become slimmer, whereas a substantial portion of the female pupal head horn disappears to form small three protrusions (S1 Fig). We tested whether *Tdic-dsx* regulates the above sexually dimorphic morphogenetic processes by injecting dsRNA targeting *Tdic-dsx* into male larvae at several developmental stages (S3 Table). As previously reported, male head horns became shorter, and thoracic horns were not formed in adults when *Tdic-dsx* dsRNA was injected at sufficiently early timepoints [8]. In these conditions, the sizes of the pupal head horns and the pupal thoracic horns were also smaller proportionally to that of shortened adult horns (-85 h APF) (Fig 7B, S4 Fig). On the other hand, males treated with *Tdic-dsx* dsRNA at later stages (13 h APF) formed thickened adult thoracic horns similar to a pupal thoracic horn before remodeling (Fig 7C) and the head horn was comparable with the wild type head horn (S4 Fig). These data indicate that DsxM is required for horn remodeling only in the thorax, and is dispensable in the head horn remodeling.

## Discussion

Here, we have described the development of sexually dimorphic horns in a Japanese rhinoceros beetle, *T*. *dichotomus*, from both morphological and genetic points of view. We focused on *Tdic-dsx* as the major regulatory gene for sexually dimorphic horn formation and investigated its time of action through expression analysis and loss-of-function analysis of sex determination genes in multiple developmental timepoints during the horn morphogenesis period. To manipulate sex-specific alternative splicing of *Tdic-dsx*, we identified *Tdic-tra* as a key regulatory factor for the sex-specific splicing of *Tdic-dsx*. We discuss below the genetic regulatory framework of sex determination in *T*. *dichotomus*, the detailed actions of *Tdic-dsx* in males and females to form sexually dimorphic horns, and evolutionary background realizing the exaggerated horn formation in horned beetles.

### Genetic regulatory mechanisms of sex determination in *T*. *dichotomus*

As mentioned in the Introduction section, the function of regulatory factors that are supposed to bind directly to *dsx* transcripts (Tra, Tra2) or Dsx protein (Ix) are conserved in many holometabolous insects. Our loss-of-function analysis data suggest that the genetic regulatory mechanisms of sex determination in *T*. *dichotomus* follow this framework.

The conserved function of Tdic-Tra and Tdic-Tra2 as splicing factors targeting *Tdic-dsx* seems to be conserved (Fig 3C). In addition, the biological functions of these genes are at least similar to those in other beetles because the RNAi phenotypes of *tra* and *tra2* orthologs in *T*. *dichotomus* (i.e. the viable masculinized phenotype in *Tdic-tra* RNAi females, and the lethal phenotype in *Tdic-tra2* RNAi females) were comparable with those reported in other beetles (*T*. *castaneum*; [40, 41], the stag beetle *Cyclommatus metallifer*; [49]).

In *D*. *melanogaster*, Ix directly binds to DsxF in females but not to DsxM in males, and functions as a co-activator to facilitate transcription activity of target genes of DsxF [32]. The intersexual phenotype observed only in *Tdic-ix* RNAi-treated females (Fig 3A) implies that Tdic-Ix might also interact with Tdic-DsxF to regulate female-specific sex differentiation as in *D*. *melanogaster*. A female-specific intersexual transformation phenotype reported in the stag beetle *C*. *metallifer* [49] suggests that the female-specific function of *ix* may be conserved among Polyphaga as well.

In contrast, our RNAi experiments implied that Tdic-Sxl did not regulate the sex-specific alternative splicing of *Tdic-tra* in females (Fig 3), which is a direct regulatory target of Sxl in *D*. *melanogaster* females. Because the splicing of *tra* orthologs are not regulated by *Sxl* orthologs even in other Dipteran species [50, 51], the sex-specific alternative splicing of *tra* may be regulated by unknown species-specific factors other than *Tdic-Sxl*.

**Fig 8:**
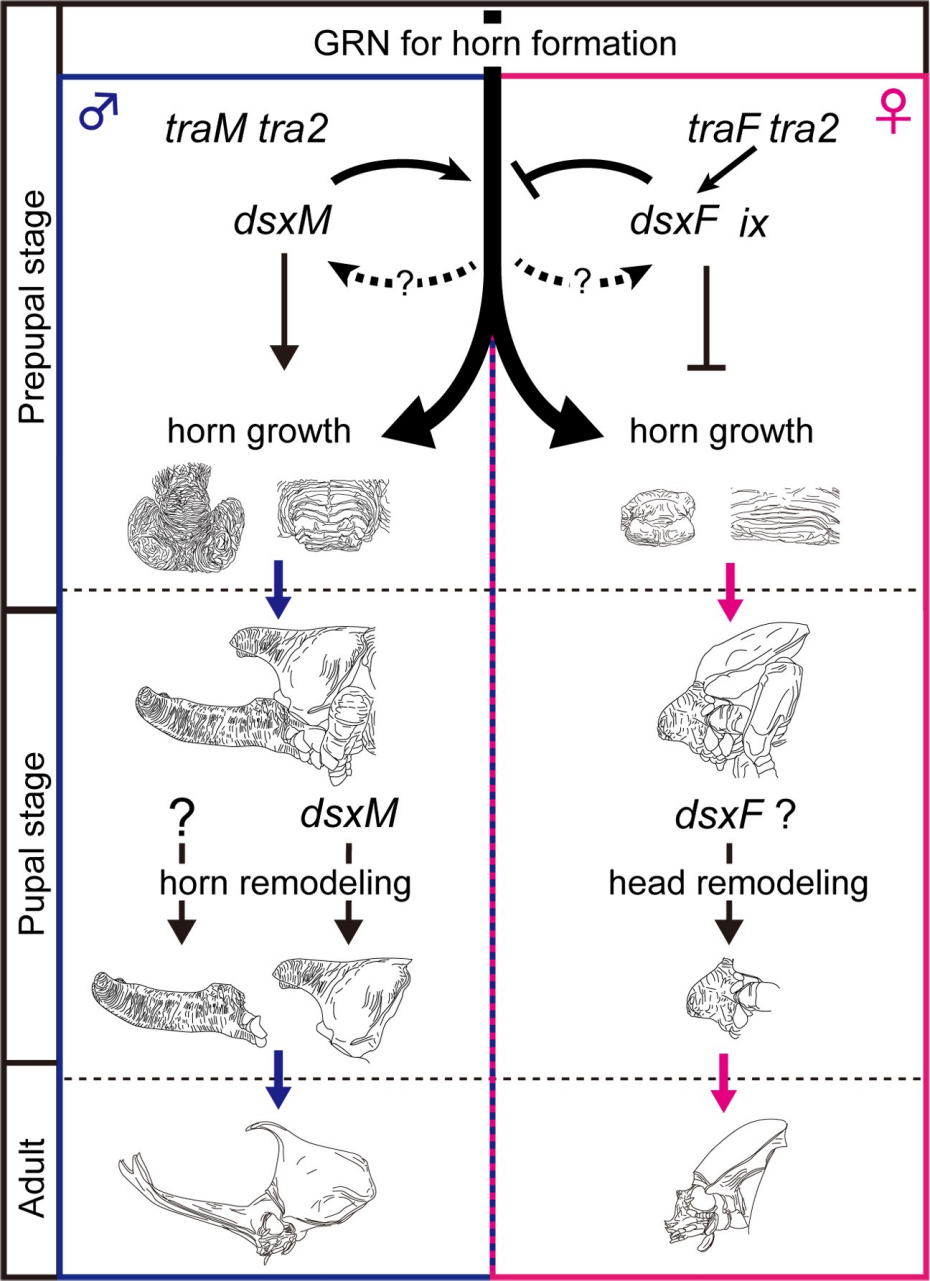
A regulatory model for the formation of sexually dimorphic horns in *T*. *dichotomus*. In males, default splicing generates *Tdic-dsxM*, whereas in females, *Tdic-tra* (a female isoform, *traF*) and *Tdic-tra2*, generates *Tdic-dsxF* by female-specific splicing of *Tdic-dsx* pre-mRNA. Prepupal *Tdic-dsxM* expression in males positively regulates the growth of head and thoracic horn primordia, whereas prepupal *Tdic-dsxF* in females suppresses the horn growth activity. In pupa, *Tdic-dsxM* regulates thoracic horn remodeling, but is dispensable for remodeling of head horn. GRN, gene regulatory network.

To summarize the regulatory mechanisms of *T*. *dichotomus* sex determination discussed above, functional Tdic-Tra is first expressed female-specifically through species-specific unknown mechanisms. Then, as in many other holometabolous insects, a Tdic-Tra/Tdic-Tra2 heterodimer is formed only in females and produces *Tdic-ds*x*F* mRNA and Tdic-DsxF to promote female differentiation by interacting with Tdic-Ix (Fig 8). On the other hand, in males, functional Tdic-Tra is not expressed, and default splicing of *Tdic-dsx* would result in *Tdic-dsxM* mRNA and Tdic-DsxM production to promote male differentiation (Fig 8).

### The gene regulatory network driving horn dimorphism formation in *T*. *dichotomus*

A previous study revealed that *Tdic-dsx* RNAi in both males and females does not result in a total loss of horns, but results in intermediate-lengthened head horn formation and loss of a thoracic horn (Fig 3) [8]. The head horn phenotype suggests that there exists a GRN for head horn formation that is operated independently of *Tdic-dsx*. *Tdic-dsxM* seems to enhance this GRN whereas *Tdic-dsxF* seems to suppress it. On the other hand, the thoracic horn phenotypes suggest that thoracic horn formation seems to be totally dependent on *Tdic-dsxM* function, and that action of *Tdic-dsxM* on horn formation might be different between head and thoracic horns during prepupa and/or pupa. However, in the course of our *Tdic-dsx* RNAi experiments, we noticed that thoraces of severely intersexually transformed individuals are always slightly bulged at the thoracic horn formation region (Fig 3A). This implies that *Tdic-dsxF* expression in the female thorax still has suppressive activity against the GRN for thoracic horn formation. If this is the case, the regulatory relation between *Tdic-dsx* and GRN for horn formation has partial similarity between the head and the thorax. The phenotypic difference previously described in head and thoracic horns would be due to difference in length of horn formed in each body region. In the sections below, we mainly discuss the GRN for horn formation based on our head horn data in which interpretation of phenotypes is less ambiguous.

Our knockdown experiment of *Tdic-tra* revealed that the onset of *Tdic-dsx* modulating GRN for head horn formation is as early as 29 h APF (Fig 4). This is approximately 7 hour before the appearance of sexual dimorphism of head horn primordium (Fig 1B). Our qRT-PCR and immunohistochemistry analysis focusing on Tdic-Dsx were consistent with this estimation: transcription and translation of *dsx* was initiated before 0 h APF, but nuclear translocation of Dsx was initiated betweeen 12 h and 36 h APF (Fig 5, S3 Fig, S5 Fig). Therefore, *Tdic-dsx* seems to modulate head horn primordium formation just before the onset of tissue growth, and following tissue morphogenesis during prepupal stages. Several genes involved in the GRN for horn formation have been identified in horned beetle species, primarily by focusing on *Drosophila* appendage patterning genes [52–54]. These studies propose an attractive evolutionary model in which large portions of the GRN for proximodistal appendage patterning were recruited to acquire beetle horns in the head and thoracic regions. Still, the overall framework of GRN for horn formation including the key regulatory gene sets involved, spatiotemporal expression dynamics, and regulatory relation among those genes, has not been unveiled so far. Therefore, investigating regulatory relations between appendage patterning genes and other head/thoracic patterning genes expressed in horn primordia focusing on this developmental timepoint will lead to verification of this model in the future studies. In addition, because candidates of the *dsx* target genes involved in GRN for horn formation were identified recently in a horned beetle, *O*. *taurus* [55], functional analyses focusing on orthologs of those genes expressed in *Trypoxylus* horn primordia at this timepoint will also lead to understanding of conserved and divergent aspects of sexually dimorphic horn formation in horned beetles.

Region-specific upregulation of *dsx* is another essential feature to drive GRN for sexual dimorphism formation in insects [17–21]. Intense studies focusing on regulatory mechanisms of sex comb formation in *D*. *melanogaster* revealed that such region-specific upregulation of *dsx* is mediated by a positive feedback loop between *dsx* and a Hox gene, *Sex combs reduced* [18]. Therefore, region-specific upregulation of *Tdic-dsx* observed in a head horn primordium might reflect an analogous positive feedback loop mechanism (Fig 5). Future research focusing on the patterning mechanisms of beetle horns and its interaction with *dsx* will be an important issue to discuss conserved genetic framework for sexual dimorphism formation.

Moreover, we found nuclear translocation of Tdic-Dsx at the onset of male head horn formation (Fig 5, S3 Fig). As far as we know, such a mode of regulation against Dsx during development is not reported in other insects. Understanding the molecular mechanisms underlying this phenomenon will be another direction to take in the future studies.

### *Tdic-dsx-*mediated sexually dimorphic morphogenesis in head and thoracic horn formation during larval-pupal development

As previously reported, *Tdic-dsx* RNAi from early stages resulted in short head horn formation in males and females (Fig 3A) [8]. These data indicate that during the prepupal stage *Tdic-dsxM* promotes the growth of head and thoracic horn primordium, whereas *Tdic-dsxF* suppresses growth of head and thoracic horn primordium (Fig 8, prepupal stage) [8]. On the other hand, RNAi treatments in later stages revealed distinct functions of *Tdic-dsx* in sexually dimorphic horn formation. During pupal-adult development, *Tdic-dsxM* regulates remodeling of a thoracic horn primordium from a rounded shape to a slender hooked shape (Fig 7, Fig 8, pupal stage), but is dispensable for head horn remodeling (S4 Fig). These results indicate that *Tdic*-*dsxM* and *Tdic-dsxF* regulate different aspects of morphogenesis at both prepupal and pupal stages. Furthermore, to what extent *Tdic-dsx* is required during larval-pupal development is also finely regulated during male head horn formation, male thoracic horn formation, female head protrusion formation, and female flat prothorax formation. Importantly, in contrast to the previous study that suggested that functions of *Tdic-dsxM* and *Tdic-dsxF* in *T*. *dichotomus* head and thoracic horn formation are to some extent analogous as described in the previous section (that is, in both of the head and the thoracic horn formation, *Tdic-dsxM* functions as a positive regulator whereas *Tdic-dsxF* functions as a negative regulator) [8], our experiments clarified that both spatial cues (i.e. different developmental contexts in head and prothorax) and temporal cues (i.e. different developmental contexts in prepupa and pupa) modulate the actions of *Tdic-dsxM* and *Tdic-dsxF* to drive appropriate morphogenetic activity in each horn primordium at each developmental stage. Since spatiotemporal modularity of gene function is often coded in modular regulatory elements in the genome (reviewed in [56]; e.g. [57–60]), the regulatory elements of *dsx* that integrates the above distinct spatiotemporal cues could be coded at the *Tdic*-*dsx* locus in the genome. Identification of such regulatory elements will be an important issue to elucidate evolution of sexually dimorphic horn formation in the future studies.

A notable feature of *Tdic-dsx* during sexually dimorphic development is that the onset of action during horn development seems to be categorized earlier (i.e. during the prepupal period) than that of many other sexually dimorphic adult traits in holometabolous insects reported so far (i.e. the pupal period). In *D*. *melanogaster* sex comb formation, *Papilio* mimetic wing morph formation, and *Bicyclus* wing pheromone gland formation, tissue-specific high expression of *dsx* orthologs are detected after pupation [18–21]. Such a difference in *dsx*’s onset of action seems to be due to the requirement of drastic growth during sexually dimorphic structure formation. In either case mentioned above, the finally formed structure accompanies little to no tissue-level drastic growth during development. On the other hand, as in *T*. *dichotomus* horn formation, *D*. *melanogaster* genital organ formation, and *C*. *metallifer* stag beetle mandible formation, whose sexually dimorphic morphogenesis is regulated by *dsx*, accompanies drastic sexually dimorphic growth during larval-pupal development [17, 61–63], and its onset of action is as early as the prepupal stage. Association between *dsx*’s earlier onset of action and requirement of drastic growth during sexual dimorphism formation suggest that recruitment of the *dsx* function in the earlier stage may be one of the prerequisites to form structurally drastically different sexual dimorphism in insects.

The intermediate head horn formation via *Tdic-tra* RNAi in females (Fig 6A–6C) revealed that the anterior region of male head horn and female small protrusions are formed from different regions in the clypeolabrum. Such a distinct developmental origin of head horn within the clypeolabrum is also clearly demonstrated in *Onthophagus* species of dung beetles [3], in which the *dsx* orthologs regulate the formation of distinct sexually dimorphic horns in either the anterior or the posterior region of the head. Analogy of multiple horn formation regions within the clypeolabrum in distinct horned beetle species implies that clypeolabrum is a hotspot of morphological innovations in horned beetles. A comprehensive understanding of the GRN for horn formation in both *Trypoxylus* and *Onthophagus* and comparative developmental studies in the future will lead to understanding of molecular mechanisms underlying the evolutionary origin and evolvability of exaggerated horns in beetles.

Here we described the accurate developmental time course of horn primordial morphogenesis during the prepupal stage in the horned beetle *T*. *dichotomus* using a time-lapse photography system. In addition, we functionally characterized both *Tdic-tra* and *Tdic-tra2*, genes that regulate the sex-specific splicing of *Tdic-dsx*. By manipulating expression levels of *Tdic-tra* and *Tdic-dsx* during different developmental time points, and by quantifying the extent of sex transformation, we revealed the following three crucial features of *Tdic-dsx* function during the development of sexually dimorphic horn formation: (1) *Tdic-dsx* modulates the GRN for horn formation as early as 29 h APF, a timepoint which corresponds to 7 h before sexual dimorphisms of horn primordia first appears; (2) *Tdic-dsx* regulates different aspects of the tissue growth, tissue death and tissue movement of horn primordia depending on both spatial (head/prothorax) and temporal (prepupa/pupa) contexts; (3) *Tdic-dsxM* and *Tdic-dsxF* promotes the formation of outgrowth structure in distinct regions within the clypeolabrum. These findings inform our understanding of the patterning mechanisms at play during *T*. *dichotomus* horn formation, as well as provide information regarding regulatory shifts in these mechanisms during the evolution of sexually dimorphic traits in horned beetles. The present study provides a good starting point to elucidate such issues.

## Materials and methods

### Insects

We purchased *T*. *dichotomus* larvae from Loiinne (Japan), and Urakiso Tennen Kabuto no Sato (Japan). The last instar larvae were sexed as described previously [8], individually fed on humus in plastic containers, and kept at 10°C until use. Larvae were moved to room temperature at least 10 days, and reared at 28°C.

### Micro-CT analysis

A male head tissue at 24 hours after pupal-chamber formation (24 h APF) was fixed in Carnoy solution at room temperature overnight, washed in 70% ethanol and stored in 70% ethanol. The sample was rehydrated through a graded ethanol series, and stained with 25% Lugol solution [64–66] for 5 days. The stained sample was scanned using an X-ray micro-CT device (ScanXmate-E090S105, Comscantechno Co., Ltd., Japan) at a tube voltage peak of 60 kVp and a tube current of 100 μA. The sample was rotated 360 degrees in steps of 0.24 degrees, generating 1500 projection images of 992 × 992 pixels. The micro-CT data were reconstructed at an isotropic resolution of 13.3 × 13.3 × 13.3 μm, and converted into an 8-bit tiff image dataset using coneCTexpress software (Comscantechno Co., Ltd., Japan). Three-dimensional tomographic images were obtained using the OsiriX MD software (version 9.0, Pixmeo, SARL, Switzerland) and Imaris software (version 9.1, Carl Zeiss Microscopy Co., Ltd., Japan). Supplemental videos were edited using Adobe Premiere Pro CC (Adobe Systems Co., Ltd., Japan).

### Time-lapse photography

To monitor the precise time course of the morphogenetic changes of male and female horn primordia during larval-pupal development, time-lapse photography was performed at 28°C every 30 minutes until they had developed into adults using a CMOS camera (VCC-HD3300, SANYO, Co., Japan).

### Observation of horn primordia development based on stable prepupal staging

We found that the head-rocking behavior observed at the end of pupal-chamber formation can be an unambiguous developmental marker for initiation of the prepupal stage (0 h APF). Using this marker, sampling of horn primordia for staging was performed every 12 hours until they had developed into pupae (120 h APF) using time-lapse photography. Head and thoracic horn primordia were dissected out from prepupae in 0.75% sodium chloride, and fixed for 90 minutes at room temperature with 4% paraformaldehyde in phosphate buffered saline (PBS). After being washed twice in PBS, photographic images were obtained with a digital microscope (VHX-900, KEYENCE, Co., Japan).

### BLAST search for sex determination genes

We searched for orthologs of the sex determination genes in the *T*. *dichotomus* RNA-seq database (PRJDB6456) using full-length complementary DNA (cDNA) sequences of *D*. *melanogaster* genes (*Sxl*, *tra*, *tra2*, *ix*) as query sequences using the tblastn program (https://blast.ncbi.nlm.nih.gov/Blast.cgi). We evaluated orthology of those genes by performing reciprocal tblastx searches against the *D*. *melanogaster* cDNA database (r6.06) using the identified *T*. *dichotomos* genes as queries. We deposited cloned partial cDNA sequences of *T*. *dichotomus* genes at the DNA Data Bank of Japan (DDBJ)/European Molecular Biology Laboratory (EMBL)/GenBank. The accession number for *Tdic-Sxl* is LC385009, for *Tdic-tra* are LC385010—LC385012, for *Tdic-tra2* are LC385013—LC385018, and for *Tdic-ix* is LC385019.

### cDNA library construction, reverse transcription-polymerase chain reaction (RT-PCR), and sequencing

Total RNA was extracted from each of head or thoracic horn primordia in wild type and RNAi-treated individuals (*EGFP*, *Sxl*, *tra*, *tra2*, *ix*, *dsx* and *dsxF*) using TRI Reagent (Molecular Research Center, Inc., USA) according to the manufacturer’s instructions. First-stranded cDNA was synthesized with the SuperScript III Reverse Transcriptase (Life Technologies Japan Ltd., Japan) using 1 μg total RNA as a template. Primer sets for cloning and double stranded RNA (dsRNA) synthesis were designed based on the cDNA sequences identified above (S1 Table). PCR was performed using Ex Taq DNA polymerase (Takara Bio Inc., Japan) according to the manufacturer’s protocol. Amplified PCR products were purified using MagExtractor (TOYOBO, Co., Ltd., Japan), and subcloned into the pCR4-TOPO vector using the TOPO TA cloning Kit (Life Technologies Japan Ltd., Japan). Sequences of the inserts were determined by a DNA sequencing service at FASMAC Co. Ltd., Japan.

### Gene expression analysis by quantitative RT-PCR (qRT-PCR)

Primer sets for qRT-PCR were designed using Primer3Plus program (http://primer3plus.com/cgi-bin/dev/primer3plus.cgi) (S1 Table). qRT-PCR was performed using the cDNA libraries synthesized above and THUNDERBIRD SYBR qPCR Mix (TOYOBO, Co., Ltd., Japan) according to the manufacturer’s instructions. The relative quantification in gene expression was determined using the 2^-ΔΔCt^ method [67].

### Larval RNAi

Primer pairs for dsRNA synthesis were designed within the common regions shared by male and female isoforms (Fig 2A, S1 Table). Partial sequences of the target sequences were amplified by PCR using primers flanked with the T7 promoter sequence in the 5’-ends. DsRNAs were synthesized using AmpliScribe T7-Flash Transcription Kit (Epicentre Technologies, Corp., USA). The purified PCR products were used as templates. Injections of dsRNA were performed as described previously [8]. DsRNA was injected into each late last instar larva or prepupa under the conditions described in S2 Table and S3 Table. *Enhanced green fluorescent protein* (*EGFP*) dsRNA was injected as a negative control.

### Horn and body length measurement

Horn length was measured by extracting the contour of a horn in the lateral view using the SegmentMeasure plugin for ImageJ 64 developed by Hosei Wada. We defined a body length as the length between the anterior tip of the clypeus in the head to the posterior most region of the abdomen, and was directly measured using a digital caliper (DN-100, Niigata seiki, Co., Ltd., Japan). Relative horn length was calculated by dividing the horn length by the body size in each RNAi treated individual, and standardized by dividing by the mean horn length of the *EGFP* RNAi-treated males. Differences of medians between treatment groups were evaluated with Brunner-Munzel test using the R package "lawstat" (ver. 3.2). These p-values were adjusted by the Bonferroni correction.

### Preparation of the anti-Tdic Dsx polyclonal antibody

A DNA fragment encoding the N-terminal region of Tdic-Dsx (1–139 a.a.) was amplified with the primer flanked with an NcoI restriction site 5′-CCATGGCCGACTCGCAAGAGTACGAAGCCA-3′, and the primer flanked with BamHI restriction site 5′-GGATCCTTAGTTATTACCAACGGTTTCCCG-3′ (each restriction site is underlined), and inserted into each of two vectors, pET-32b and pET-41b (Merck KGaA., Germany), in order to express Trx- and GST-fused recombinant proteins (Trx–Tdic-Dsx and GST–Tdic-Dsx) in *Escherichia coli*, BL21(DE3*)* (New England Biolabs, Inc., Japan). Trx–Tdic-Dsx and GST–Tdic-Dsx were recovered from SDS polyacrylamide gel after electrophoresis, and stored at -20°C until use. Guinea pig polyclonal antibodies were raised against the GST–Tdic-Dsx, and affinity-purified with a Hitrap NHS-activated HP column (GE Healthcare UK Ltd., UK) coupled with the above Trx–Tdic-Dsx fusion protein.

### Immunohistochemistry

After dissection, head horn primordia fixed with 4% PFA was embedded in SCEM compound (Leica Microsystems, GmbH., Japan) and stored at -80°C until use. The frozen block was sectioned transversely at 10 μm using Leica CM1950 (Leica Microsystems, GmbH., Japan), and washed with PBS after brief drying. Guinea pig anti-Tdic-Dsx antibody was used as the primary antibody at a 1:500 dilution. Biotin-labeled goat anti-guinea pig IgG antibody (Jackson ImmunoResearch Laboratories, Inc., USA) was used as the second antibody at a 1:500 dilution. For fluorescence staining, streptavidin-HRP and Cy3-conjugated tyramide (PerkinElmer Japan Co., Ltd., Japan) were used at dilutions of 1:5000, 1:500, respectively. Laser scanning confocal microscopy (Olympus FV-1000) was used to visualize immunostained frozen tissue sections. Tiled array images were obtained using a confocal microscopes equipped with a motorized stage (Olympus 3D mosaic imaging system).

## Supporting information

S1 Fig Morphological comparison of pupal horn primordia and adult horns in *T*. *dichotomus*. Pupal horn primordia are rounded, and slightly larger than adult horns. Light blue in pupal horn primordia shows the morphologies of adult male horn or female three small protrusions.
(TIF)

S2 Fig The relative *Tdic-Sxl* expression level in *Tdic-Sxl* RNAi males and females. *Tdic-Sxl* mRNA expression levels in *Tdic-Sxl* RNAi males and females were quantified by qRT-PCR. The expression levels of *Tdic-Sxl* were decreased with *Tdic-Sxl* RNAi males and females.
(TIF)

S3 Fig High magnification images of another area in Fig 5. The head epidermis including the head horn primordium was stained with DAPI (magenta) to label nuclei and with anti-Tdic-Dsx antibody (green) to label Tdic-Dsx protein. (A) Tdic-Dsx expression pattern in 12 h APF. (B) Tdic-Dsx expression pattern in 36 h APF.
(TIF)

S4 Fig Head horn formation phenotypes induced by *Tdic-dsx* RNAi at late prepupal stages. Head horn remodeling during pupal-adult development in males. (A, A’) A wild type male head horn. (B, B’) Head horn formed by a late *Tdic-dsx* RNAi treatment (-13 h APF). (C, C’) Head horn formed by an early *Tdic-dsx* RNAi treatment (-85 h APF). *Tdic-dsxM* is dispensable for head horn remodeling. (A)–(C) the lateral views of a head horn, (A’)–(C’) the dorsal views of a head horn tips. Scale bars are 5 mm.
(TIF)

S5 Fig Time course of relative *Tdic-dsx* mRNA expression level. mRNA expression levels at each timepoint were quantified by qRT-PCR. *Tdic-dsx* was highly expressed from 24 h APF.
(TIF)

S1 Table Primers used in this study. (PDF)

S2 Table RNAi treatment conditions in Fig 3 and Fig 6. (PDF)

S3 Table RNAi treatment conditions in Fig 4, Fig 7 and S3 Fig. (PDF)

S1 Movie Micro-CT analysis of a male prepupal head at 24 h APF showing head horn primordium formation region. Head horn primordium formation regions within clypeolabrum. A small epidermal protrusion in the clypeolabral, which seems to be a head horn primordium, was observed above a clypeal primordium (Fig 1A). The head capsule is indicated in blue [44].
(MP4)

S2 Movie Time-lapse photography of last instar larvae. A head-rocking behavior starts to be observed at the end of pupal chamber formation.
(MP4)

S3 Movie Micro-CT analysis of a male prepupal head at 24 h APF showing the morphological character of the mandibular apodeme. The morphological character of the mandibular apodeme at 24 h APF. Apolysis was incomplete in the ocular and the mandibular apodeme at 24 h APF [44–46]. After this stage, prepupal horn primordia can be readily dissected out due to apolysis at the ocular and the mandibular apodeme (Fig 1B). This morphological character is unambiguous developmental marker to know the onset of sexual dimorphism formation (36 h APF).
(MP4)
