# Supplementary figures and images for: Precise staging of beetle horn formation in Trypoxylus dichotomus reveals the pleiotropic roles of doublesex depending on the spatiotemporal developmental contexts
Source: PLoS Genet. 2019 Apr 10;15(4):e1008063. doi: 10.1371/journal.pgen.1008063 (PMC6457530; doi:10.1371/journal.pgen.1008063)

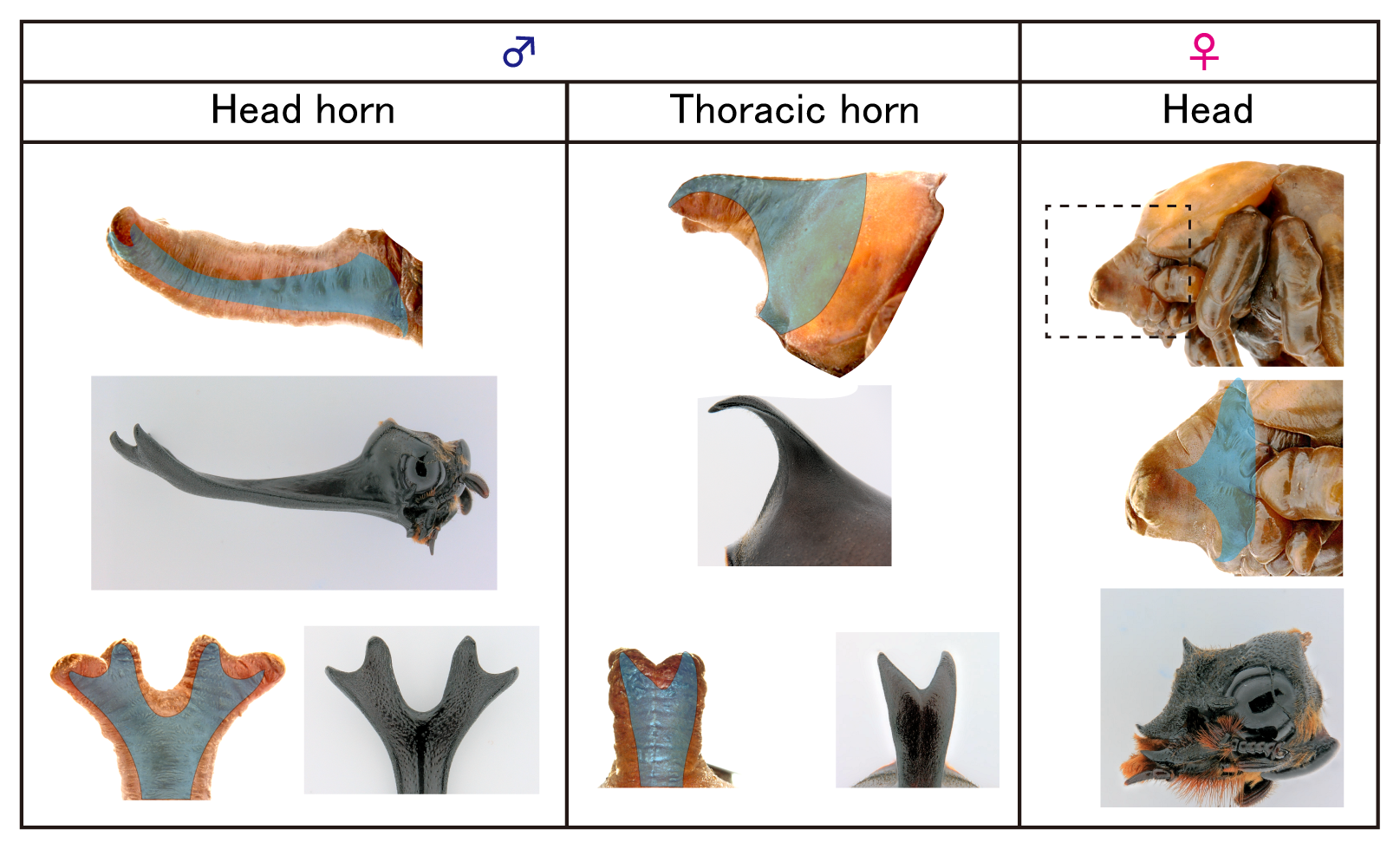

Supplement: S1 Fig — Pupal horn primordia are rounded, and slightly larger than adult horns. Light blue in pupal horn primordia shows the morphologies of adult male horn or female three small protrusions. (TIF) [file pgen.1008063.s001.tif]

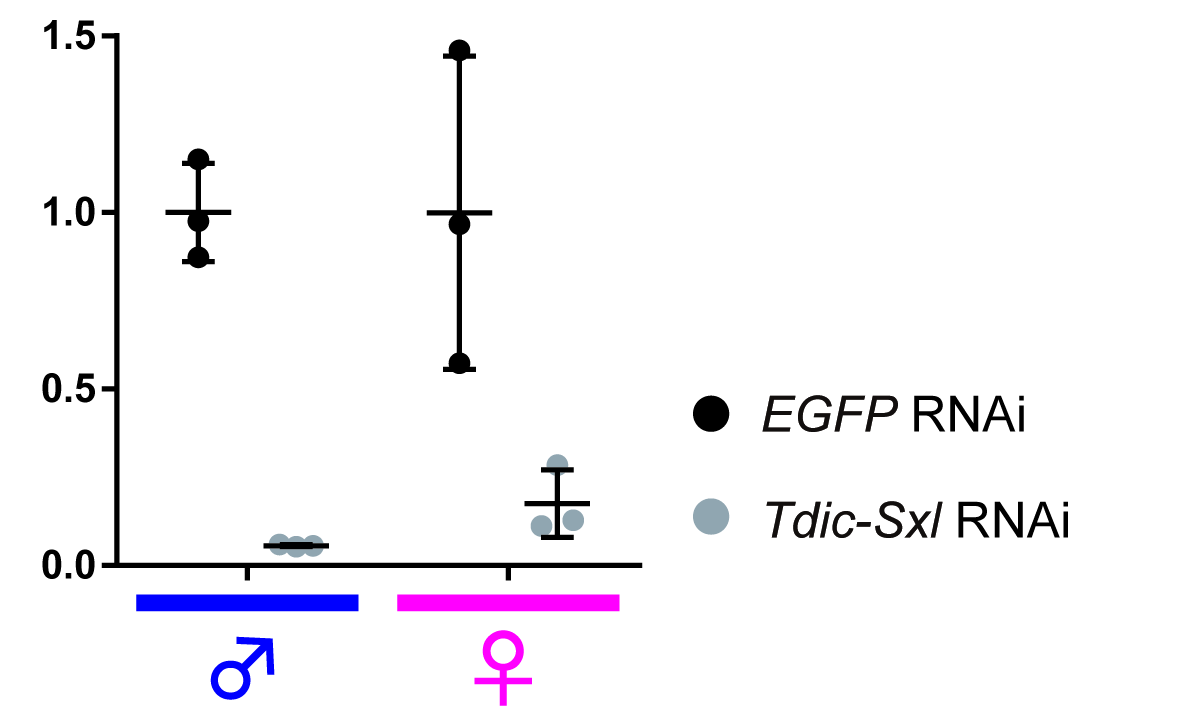

Supplement: S2 Fig — Tdic-Sxl mRNA expression levels in Tdic-Sxl RNAi males and females were quantified by qRT-PCR. The expression levels of Tdic-Sxl were decreased with Tdic-Sxl RNAi males and females. (TIF) [file pgen.1008063.s002.tif]

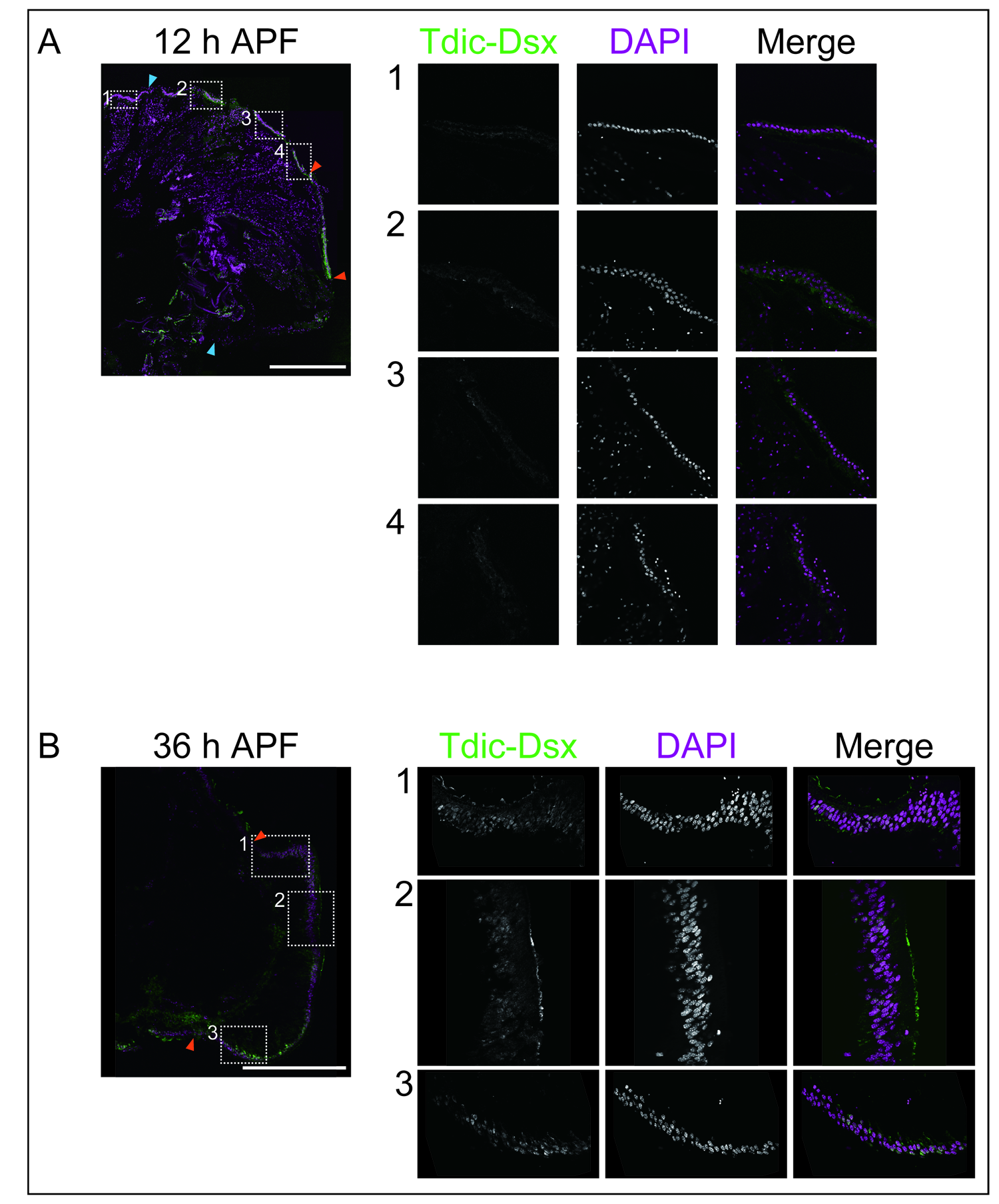

Supplement: S3 Fig — The head epidermis including the head horn primordium was stained with DAPI (magenta) to label nuclei and with anti-Tdic-Dsx antibody (green) to label Tdic-Dsx protein. (A) Tdic-Dsx expression pattern in 12 h APF. (B) Tdic-Dsx expression pattern in 36 h APF. (TIF) [file pgen.1008063.s003.tif]

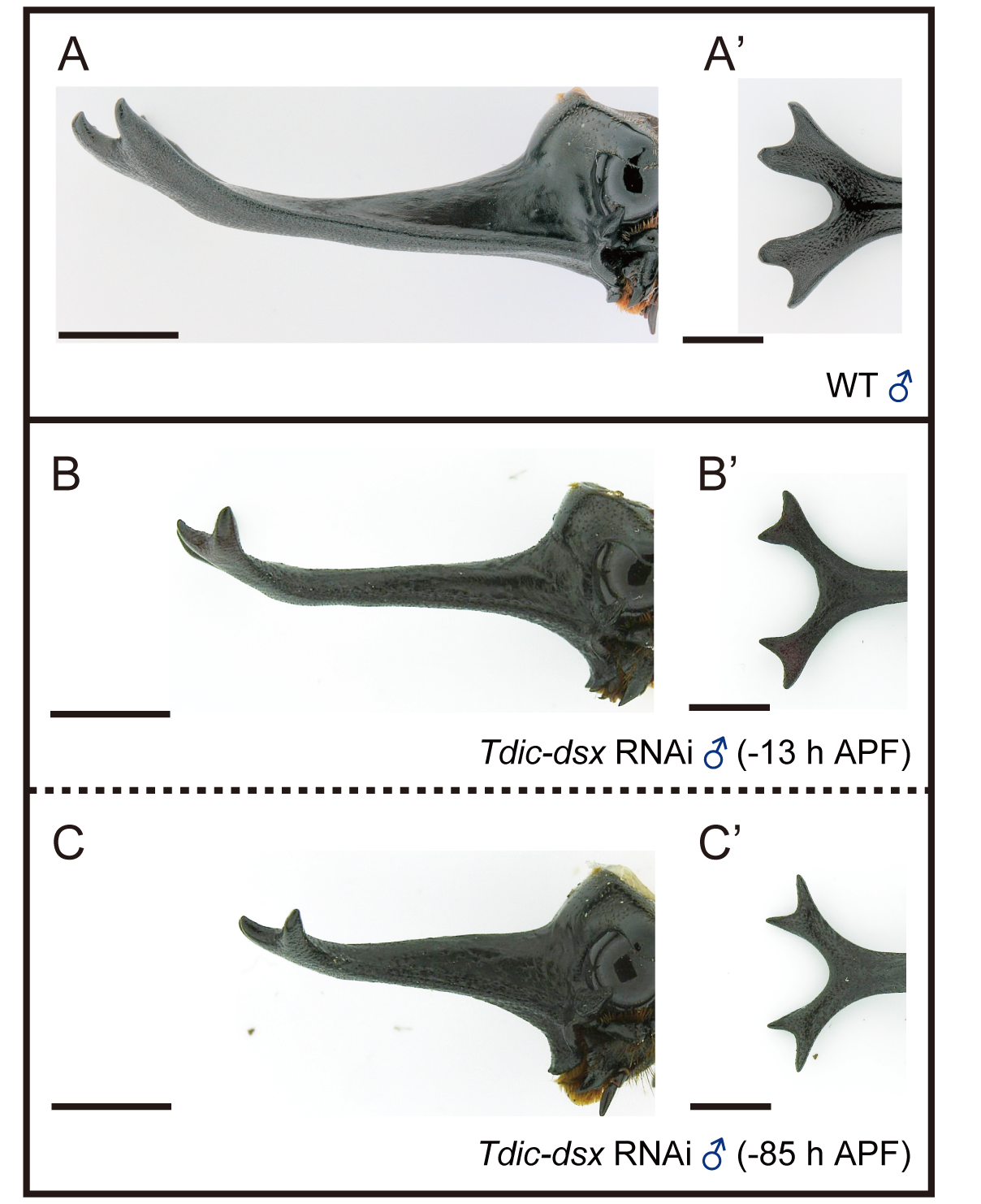

Supplement: S4 Fig — Head horn remodeling during pupal-adult development in males. (A, A’) A wild type male head horn. (B, B’) Head horn formed by a late Tdic-dsx RNAi treatment (-13 h APF). (C, C’) Head horn formed by an early Tdic-dsx RNAi treatment (-85 h APF). Tdic-dsxM is dispensable for head horn remodeling. (A)–(C) the lateral views of a head horn, (A’)–(C’) the dorsal views of a head horn tips. Scale bars are 5 mm. (TIF) [file pgen.1008063.s004.tif]

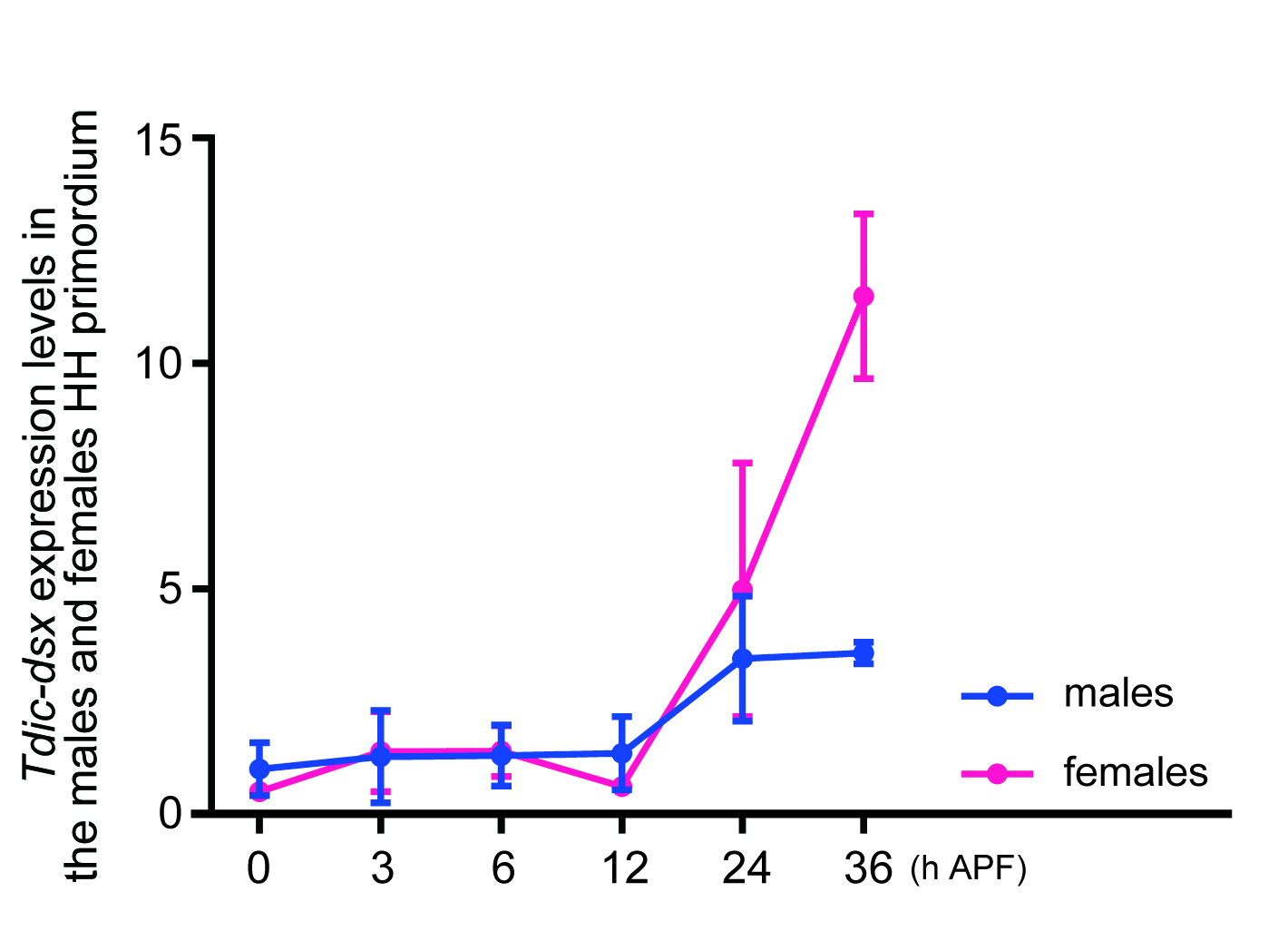

Supplement: S5 Fig — mRNA expression levels at each timepoint were quantified by qRT-PCR. Tdic-dsx was highly expressed from 24 h APF. (TIF) [file pgen.1008063.s005.tif]
