# Supplementary material for: Precise staging of beetle horn formation in Trypoxylus dichotomus reveals the pleiotropic roles of doublesex depending on the spatiotemporal developmental contexts
Source: PLoS Genet. 2019 Apr 10;15(4):e1008063. doi: 10.1371/journal.pgen.1008063 (PMC6457530; doi:10.1371/journal.pgen.1008063)
Supplement: S1 Table — (PDF) [file pgen.1008063.s006.pdf]

S1 Table. Primers used in this study.

	Gene	Forward (F) / Reverse (R)	Sequence (5' to 3')
Clonning	*Tdic-Sxl*	F	taatacgactcactatagggATGTTCGCTAATAAACG
		R	CTAATCATGCTGACAATATTGGC
	Tdic-tra	F	ATGAGTAAGAGACTTCCACC
		R	CTAATCAGTAGTTTTAATGATCTCTTG
	Tdic-tra2	F	CCGCATACTATGAGTGATAGAGAG
		R	TCATCCAATACCACGTGTTC
	Tdic-ix	F	ATGAATGTTGTTGGTATGGG
		R	TCATTCTGATGGTGATATATTC

	Gene	Forward (F) / Reverse (R)	Sequence (5' to 3')
dsRNA	*EGFP*	F	taatacgactcactatagggAATCATGGCCGACAAGCAGAA
		R	taatacgactcactatagggAAACTCCAGCAGGACCATGTG
	Tdic-Sxl	F	taatacgactcactatagggATGTTCGCTAATAAACG
		R	taatacgactcactatagggTCGATTGGGCCGATCTGTGAG
	Tdic-tra	F	taatacgactcactatagggAAGGGTTGTGGTTGCTACG
		R	taatacgactcactatagggCCGGCGAGATCTTCTATCTG
	Tdic-tra2	F	taatacgactcactatagggTCGCAGGCGCCACATGGGCA
		R	taatacgactcactatagggTCTCTGTCTCTATCTCGGCG
	Tdic-ix	F	taatacgactcactatagggAGCAACCTCAAGCATTGGAT
		R	taatacgactcactatagggGTGCATTTGCAGTCGCTAAA

	Gene	Forward (F) / Reverse (R)	Sequence (5' to 3')
qRT-PCR	*Tdic-Sxl*	F	ATTACTGGGTTGCCGAGGGG
		R	GTCCAAGTTCGCGATGGCAG
	Tdic-tra	F	ATCAAAGAAGGCCTCCGCGT
		R	ATCTAGGCGTTGGAGCAGGC
	Tdic-tra2	F	GTACAGCCATCGCAGATCGC
		R	CACGTGTTCTTAACCGTGGTGA
	Tdic-ix	F	GCAACAGCCTCAGCAACCTC
		R	CACAATCAAGACTGCAGCGC
	Tdic-dsx	F	TAGTTAGTGATGTCCGACTCGCA
		R	GTTCGGGGATTCGTCGACGT
	Tdic-dsxF	F	AGGCAACCCTTACGTCTGTCA
		R	ACGATTTCTTCATGTGGCGCA
	Tdic-RpL32	F	AAGACACGCCATATGCTTCC
		R	CACCATGTGCAATCTCTCCA

	Gene	Forward (F) / Reverse (R)	Sequence (5' to 3')
RT-PCR	*Tdic-Sxl*	F	taatacgactcactatagggATGTTCGCTAATAAACG
		R	CTAATCATGCTGACAATATTGGC
	Tdic-tra	F	ATGAGTAAGAGACTTCCACC
		R	TTCGGACGTAGCAACCACAACC
	Tdic-tra2	F	CCGCATACTATGAGTGATAGAGAG
		R	AGCTCGTCCTCGGTGGTGTAGACGC
	Tdic-ix	F	ATGAATGTTGTTGGTATGGG
		R	TCATTCTGATGGTGATATATTC
	Tdic-dsx	F	TATGCCATTATGAAGCTAGCGCC
		R	TATGACTTGGACTAATGAGATCATC
	Tdic-RpL32	F	CTGACCGTTATGGAAAACTT
		R	TACGATTTTGCATCAACAAT

T7 promoter sequence are written in lowercase
