# Supplementary material for: Precise staging of beetle horn formation in Trypoxylus dichotomus reveals the pleiotropic roles of doublesex depending on the spatiotemporal developmental contexts
Source: PLoS Genet. 2019 Apr 10;15(4):e1008063. doi: 10.1371/journal.pgen.1008063 (PMC6457530; doi:10.1371/journal.pgen.1008063)
Supplement: S2 Table — (PDF) [file pgen.1008063.s007.pdf]

S2 Table. RNAi treatment conditions in Fig 3 and Fig 6.

dsRNA	Sex	Injected dsRNA (μg)	Number of injected larvae	Eclosed to adult	Number of adults with sex specific phenotypes	Efficiency(%)
EGFP	♂	10	8	6	0	0
		20	8	7	0	0
	♀	10	8	7	0	0
		20	8	8	0	0
Tdic-Sxl	♂	2.5	8	2	0	0
		5	8	4	0	0
		10	8	3	0	0
		15	8	4	0	0
		20	8	6	0	0
		50	8	7	0	0
	♀	2.5	8	7	0	0
		5	8	5	0	0
		10	8	3	0	0
		15	8	6	0	0
		20	8	7	0	0
Tdic-tra	♂	2.5	8	5	0	0
		5	15	9	0	0
		10	10	7	0	0
		15	8	5	0	0
	♀	2.5	8	5	5	100
		5	23	17	15	88
		10	12	8	7	88
		15	8	5	5	100
Tdic-tra2	♂	0.5	8	2	0	0
		1	18	0	0	-
		5	18	0	0	-
		10	18	0	0	-
	♀	0.5	8	0	0	-
		1	18	0	0	-
		5	18	0	0	-
		10	18	0	0	-
Tdic-ix	♂	5	13	11	0	0
		10	17	15	0	0
		15	8	8	0	0
		20	8	8	0	0
	♀	5	20	15	14	93
		10	18	14	14	100
		15	8	6	6	100
		20	8	5	5	100
Tdic-dsx	♂	15	8	5	5	100
	♀	15	8	6	6	100
