# Supplementary material for: Precise staging of beetle horn formation in Trypoxylus dichotomus reveals the pleiotropic roles of doublesex depending on the spatiotemporal developmental contexts
Source: PLoS Genet. 2019 Apr 10;15(4):e1008063. doi: 10.1371/journal.pgen.1008063 (PMC6457530; doi:10.1371/journal.pgen.1008063)
Supplement: S3 Table — (PDF) [file pgen.1008063.s008.pdf]

S3 Table. RNAi treatment conditions in Fig 4, Fig 7 and S3 Fig.

dsRNA	Sex	Injected dsRNA (μg)	Number of injected prepupa	Eclosed to adult
EGFP	♂	15	25	20
	♀	15	35	26
Tdic-tra	♀	15	91	63
Tdic-dsx	♂	15	43	29
